# Multi‐Tool Marine Metabarcoding Bioassessment for Baselining and Monitoring Species and Communities in Kelp Habitats

**DOI:** 10.1111/1755-0998.70010

**Published:** 2025-07-17

**Authors:** Giulia Maiello, Marilla R. Lippert, Erika F. Neave, Erik A. Hanson, Stephen R. Palumbi, Stefano Mariani

**Affiliations:** ^1^ School of Biological and Environmental Sciences Liverpool John Moores University Liverpool UK; ^2^ Hopkins Marine Station, Department of Biology Stanford University Pacific Grove California USA; ^3^ Department of Life Sciences Natural History Museum London UK

**Keywords:** biomonitoring, environmental DNA, kelp forests, marine communities, marine protected areas

## Abstract

The astonishing biological diversity found in Californian kelp forests requires efficient and robust monitoring tools to better understand ecological trends and mitigate against loss or disruption of ecosystem services due to human pressure and climate changes. With environmental DNA (eDNA) metabarcoding becoming a popular biodiversity assessment approach, we set out to evaluate a combination of powerful, rapid and sustainable eDNA solutions for characterising marine community composition in kelp‐dominated habitats along the central California coast, in the newly proposed Chumash Heritage National Marine Sanctuary. We employed and compared the efficiency of several eDNA collection approaches, including ‘traditional’ surface water filtration, the collection of organisms encrusting cobble rocks and various deployments of an artificial passive sampler, the metaprobe (i.e., attached to divers, dangled from a boat and cast from the shore using a fishing rod). By combining the information from fish specific (Tele02 12S) and universal metazoan (COI) markers, we ‘captured’ 501 unique marine taxa, belonging to at least 36 phyla, over 400 of which were identified to genus/species level, and including 52 vertebrate species typical of Californian kelp forest ecosystems. Despite differences in the type of biodiversity returned by the tested sampling methods, the overall community structure of the surveyed area was highly spatially structured and strongly influenced by the biogeographic break around Point Conception (*Humqaq*). We discuss the benefits of integrating eDNA metabarcoding in existing monitoring programs and devising a reproducible approach to monitor faunal changes in kelp forest habitats and beyond.

## 1 Introduction

Human impacts and climate change are altering the chemical and physical characteristics of coastal waters with dramatic consequences on marine biodiversity (He and Silliman 2019). Broad‐scale biomonitoring of marine ecosystems, especially coastal and shelf habitats, is crucial for the early detection of biological changes (Dafforn et al. 2016) and requires rapid, efficient and robust monitoring tools to better understand the processes and rate at which assemblage structure and ecosystem functions are being transformed.

Traditional assessment methods based on the visual taxonomic identification of captured specimens can be laborious, environmentally impactful and require skilled taxonomists to identify diverse groups across the tree of life, yet still can fail to comprehensively portray biodiversity features of large and complex ecosystems (Hebert et al. 2003). DNA metabarcoding overcomes many of the limitations of morphology‐based biomonitoring, as it combines the use of universal primers with next‐generation sequencing (NGS) to document multispecies assemblages across the tree of life, including the discrimination of cryptic species and visually indistinguishable life stages such as larvae and eggs (Bessey et al. 2020). The agility and accuracy of environmental DNA (eDNA) metabarcoding offers new opportunities for upscaling data collection (Gilbey et al. 2021). It can detect a greater number of species across multiple trophic and taxonomic levels (Kelly et al. 2017; Stat et al. 2017) than traditional methods and covers broader spatial and temporal scales (Thomsen and Willerslev 2015; Sales et al. 2021; West et al. 2021), which is crucial given the ongoing threats to global biodiversity.

The coast of California is characterised by rich and biologically productive marine environments, based on coastal currents and seasonal winds that generate considerable upwelling of cold and nutrient‐rich water, supporting diverse and abundant marine fauna and complex food webs (Gleason et al. 2006). Kelp forests are perhaps the most emblematic ecosystems of the California coastline, which are primarily constituted by large, fast‐growing brown algae of the order Laminariales (Dayton 1985). These habitat‐forming kelps create essential habitats for a diverse array of organisms, including marine mammals, fishes, invertebrates and other algae that collectively make this one of the most diverse and productive habitats of the planet (Mann 1973; Steneck et al. 2002).

Along the central Californian coast, the first Tribal‐nominated national marine sanctuary was designated in October 2024: the Chumash Heritage National Marine Sanctuary, which stretches along 156 miles of coastline, covering 4543 mile^2^ of water, south of the southern boundary of the Monterey Bay National Marine Sanctuary toward the northern edge of the Channel Islands National Marine Sanctuary. The proposed sanctuary encompasses three marine protected areas (i.e., Pt. Buchon, Vandenberg and Pt. Conception from north to south) and features a significant marine biogeographic break at Point Conception (*Humqaq* in Chumashan). The area is recognised as a biological hotspot for birds, marine mammals, sea turtles, fishes and other marine organisms, like kelp algae. Some biological groups are highly spatially structured around *Humqaq* (Blanchette et al. 2008; Claisse et al. 2018), including rocky reef fish (Hamilton et al. 2010), intertidal invertebrates (Blanchette et al. 2009), rocky intertidal algae (Murray and Littler 1981) and subtidal macro‐invertebrate communities (Zahn et al. 2016). Nevertheless, the overall marine community structure near the geographic shift at *Humqaq* remains poorly documented, largely due to the remoteness of the area, which includes marine protected areas with limited easy access, such as Vandenberg State Marine Reserve. Furthermore, Californian kelp forests monitoring programmes, such as PISCO annual monitoring surveys, mostly rely on visual scuba diver observations, which have several limitations for broadscale monitoring of extensive ecosystems on a wide range of taxa. Thus, there is an urgent need for the development and implementation of more efficient and robust methods to comprehensively characterise the multitaxon diversity of the region.

Molecular tools, such as eDNA metabarcoding, are well placed to generate extensive species inventories and characterise changes in species assemblages across marine biogeographic breaks (DiBattista et al. 2022; West et al. 2021). Numerous eDNA collection methods have been developed, each with its advantages and limitations. Selecting a method typically involves balancing trade‐offs among throughput, cost, speed and feasibility (Minamoto et al. 2016). The main constraint of traditional eDNA approaches is the collection and filtration of large water volumes, a time‐consuming process that requires sterile equipment and dedicated personnel. This sometimes limits the number of samples in eDNA studies (Bessey et al. 2021; Hinlo et al. 2017) and poses challenges for collection in remote locations (Hansen et al. 2020). Various artificial passive sampling strategies have been proposed to circumvent these limitations (Bessey et al. 2021; Bessey et al. 2022; Jeunen et al. 2022; Verdier et al. 2022). One such strategy is the use of the ‘metaprobe’, a low‐tech, reusable 3D‐printed sampler, which has been successfully used to collect eDNA through absorption when deployed in fishing nets (Maiello et al. 2022). With the goal of fine‐tuning an approach for long‐term biodiversity monitoring in the newly proposed Chumash Heritage National Marine Sanctuary (https://sanctuaries.noaa.gov/chumash‐heritage/) we employed and compared several methods, including ‘classical’ surface water filtration, the collection of organisms encrusting cobble rocks (Shum et al. 2019) and a range of novel deployments of the metaprobe (i.e., attached to divers, dangled from a boat and cast from the shore using a fishing rod). Samples underwent metabarcoding screening using two complementary markers, with the specific aims to: (1) compare the different DNA collection approaches in their ability to capture marine biodiversity, (2) examine differences in the type of biodiversity information returned by each of the tested sampling methods (e.g., benthic vs. pelagic and taxonomic biases) and (3) investigate the effectiveness of DNA metabarcoding data in portraying changes in marine assemblages along the Californian biogeographic cline.

## 2 Materials and Methods

### 2.1 Sampling

**FIGURE 1:**
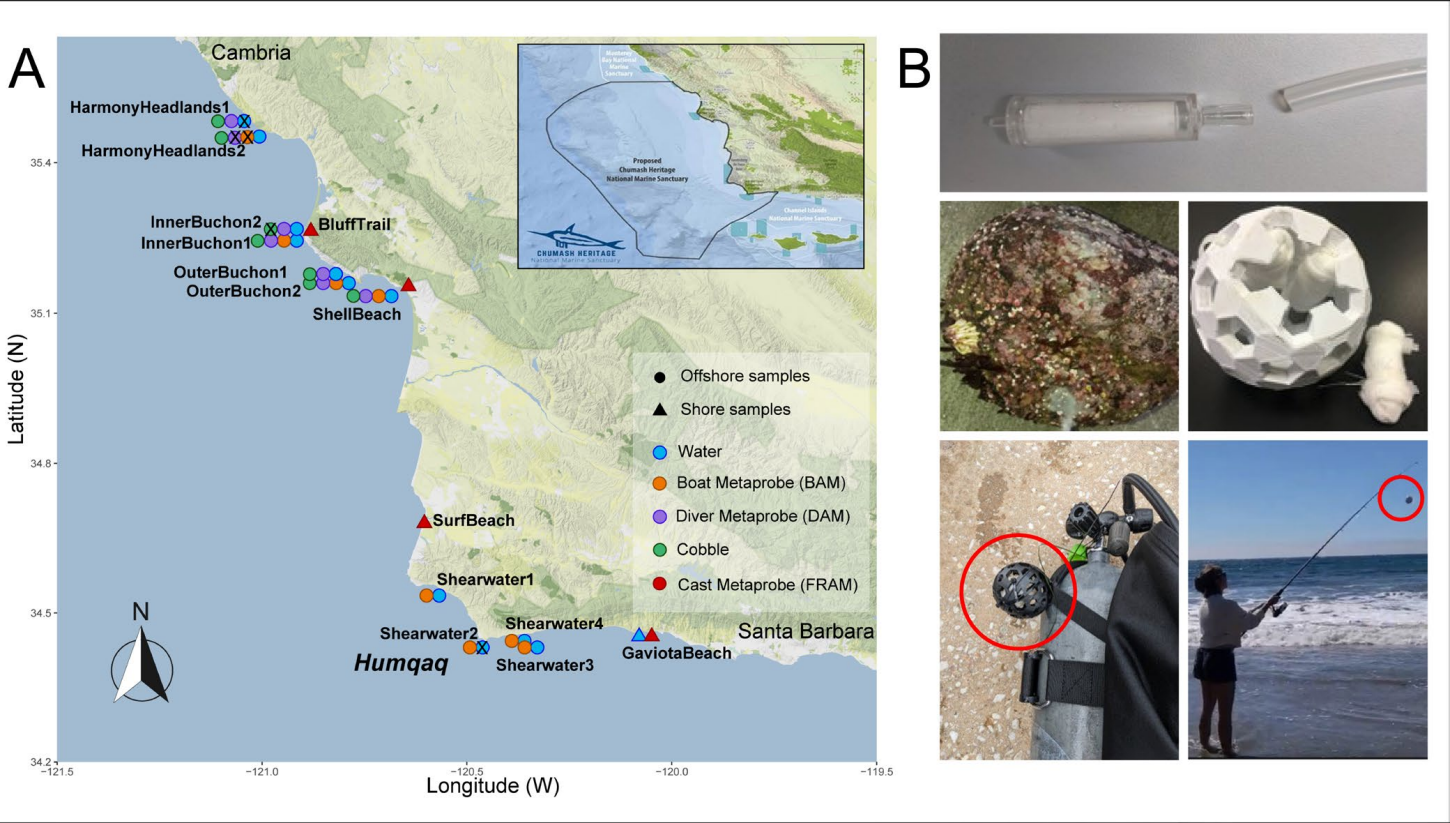
(A) Map of the 15 sampling sites along the Californian coast in the Chumash Heritage National Marine Sanctuary area. The inset at the top shows the proposed boundaries of the sanctuary between Monterey Bay and the Channel Island Marine Sanctuaries (source: National Oceanic and Atmospheric Administration (NOAA) ‐ https://sanctuaries.noaa.gov/chumash‐heritage/). For each site, circle colours represent the type of sampling method employed, while offshore and shore sites have different shapes. The ‘X’ on top on some sampling dots highlight the sampling types that were lost through sample processing. The map was created using the R‐package GGMAP (Kahle and Wickham 2013). (B) Pictures of sampling methods. From top to bottom and from left to right: Sterivex filter (source: https://github.com/boopsboops/seadna‐protocols/), cobble rock, the ‘metaprobe’ with rolls of gauze beside and inside it, the ‘metaprobe’ attached to a scuba diver's tank, the ‘metaprobe’ cast from the shore.

Environmental DNA samples were collected at the end of September 2022 from 15 sampling sites from dense kelp forest sites along the central Californian coast within and adjacent to the Chumash Heritage National Marine Sanctuary (CHNMS). Eleven sites were located just offshore on kelp beds, while the other four were situated on the shore (Figure 1; Table S1). Offshore samples were collected by boat, testing four different approaches to gathering environmental and community DNA. The first one was by ‘traditional’ water filtering (hereinafter eDNA): at each site, we collected three 1.5 L bottles of surface seawater from the stern of the boat. Water samples were immediately placed on ice, away from UV light. At the end of each day, water samples were filtered through a 0.22 μm Sterivex filter (PES membrane, Merck Millipore) using a 60 mL polypropylene syringe. The three filters were placed in separate zip‐lock bags and immediately frozen. Environmental DNA was also collected through passive absorption using the metaprobes. Metaprobes were prepared in a clean environment securing three rolls of sterile gauze to each probe using zip‐ties. During sampling procedures, metaprobes were deployed in two distinct ways: (i) diver‐assisted metaprobe (DAM), where metaprobes were attached to the scuba divers tank and submerged underwater for approximately 30–40 min in the kelp forest while divers were swimming to collect cobble rocks and (ii) boat‐assisted metaprobe (BAM), where metaprobes rigged with 1–2 oz. fishing weights were submerged from the boat down toward the seafloor for approximately 15 min using a nylon strand for retrieval. BAM samples were collected while the boat was stationary during diving activities. After metaprobes were retrieved, the three gauze sub‐samples were immediately stored in separate 50 mL falcon tubes with 70% isopropyl to preserve the DNA. Lastly, divers collected medium‐sized cobble rocks (15–20 cm diameter) from the seafloor (10 per each sampling site), which were placed in separate zip‐lock bags filled with seawater until processing at the end of each sampling day. Cobbles were then gently washed with clean brushes in the zip‐lock bags and this suspension filtered through cone filter paper (mesh size: 0.45 μm, Tong Gu) to separate bulk biological material present, and then washed into glass jars with 70% ethanol. Shore eDNA samples were collected attaching a metaprobe to a fishing rod (Fishing Rod Actualized Marine Metaprobe eDNA Retriever, FRAMMER) that was cast from the shore and submerged for approximately 10 min around kelp forest patches. Gauze rolls in the metaprobes were then retrieved and separately stored in 50 mL falcon tubes with 70% isopropyl. We collected samples using a variety of methods depending on weather conditions. At four offshore sites we collected water, cobbles, BAM and DAM samples. At three offshore sites, we collected water, cobbles and DAM samples. At the four sites located in the area south of *Humqaq*, diving was impossible due to hazardous meteorological conditions, so we collected only BAM and water samples. Shore FRAMMER samples were collected at four sites. At one site (Gaviota Beach) water was collected on shore in front of a kelp patch, as weather conditions prevented boat sailing that day (Figure 1, Table S1). For each sampling method, field controls were collected every day to monitor potential contamination linked with sampling procedures. Specifically, (1) one 1.5 L bottle of distilled water was passed through Sterivex filters alongside the seawater samples; (2) a clean metaprobe was opened on the boat and the rolls of gauze were stored in 50 mL falcon tubes with 70% isopropyl to monitor the boat background contamination noise for BAM and DAM samples; (3) 1.5 L of distilled water was filtered through the cone filter paper, as cobble sample field control; (4) one clean metaprobe was used as field control for each shore sampling site where FRAM samples were collected. All the samples were stored at −20°C in the laboratory until further processing.

### 2.2 Laboratory Procedures

We used separate laboratories for DNA extraction, pre‐ and post‐PCR procedures in order to reduce the risk of contamination. For each site, two of the three water filter and metaprobe gauze replicates were processed. Prior to DNA extraction, half of each Sterivex filter or portions from various parts of each gauze roll were cut into small pieces. The amount of gauze used for DNA extraction was adjusted to fit into a 1.5 mL Eppendorf tube. The remaining Sterivex filters and gauzes were stored at −20°C for future possible re‐extractions. Gauze pieces were dried using blotting paper to remove residual ethanol to avoid PCR inhibition. Both Sterivex filters and gauze rolls were then DNA extracted following the Mu‐DNA tissue protocol (Sellers et al. 2018). DNA was lysed overnight at 37°C with 730 μL of lysis solution (1 M Tris–HCl [pH 8], 0.5 M EDTA [pH 8]), 250 μL of soil lysis additive (180 mM aluminium ammonium sulphate dodecahydrate, 20% SDS) and 20 μL of proteinase K (100 μg/mL). We then extracted the DNA according to the main steps of the protocol: inhibitor removal, silica binding, wash and final elution. Filtered suspensions from the material washed from each cobble were extracted using the materials and protocols from the Qiagen DNeasy PowerSoil Pro kit, following Shum et al. (2019). Extraction negatives (one for each extraction day) were included to monitor potential contamination linked with extraction procedures and reagents. The isolated DNA was PCR amplified targeting two mitochondrial regions: a ~167 bp fragment of the 12S ribosomal RNA gene and a ~313 bp fragment of the cytochrome oxidase I (COI) gene. The 12S was amplified using the fish‐specific Tele02 primers (forward: 5′‐AAACTCGTGCCAGCCACC‐3′; reverse: 5′‐GGGTATCTAATCCCAGTTTG‐3′; Taberlet et al. 2018), while the COI was amplified using highly degenerate universal metazoan primers (forward mICOIintF: 5′‐GGWACWRGWTGRACWNTNTAYCCYCC‐3′ (Leray et al. 2013); reverse jgHCO2198: 5′‐TAIACYTCIGGRTGICCRAARAAYCA‐3′ (Geller et al. 2013)). Specifically, water eDNA, DAM and BAM samples were amplified using both primer sets, while cobble samples were amplified only with COI and FRAM samples only with Tele02 12S. To account for possible contamination occurring along PCR procedures, we included both a positive (*Pangasianodon hypophthalmus* for Tele02 12S and *Penaeus vannamei* for COI) and a negative control for each PCR amplification run. To unequivocally identify samples and reduce the risk of cross‐contamination and/or tag switching during sequencing, each sample was amplified using a unique dual 8 bp oligo‐tag attached to the primers. Each tag differed by at least three base pairs from all other tags and was preceded by two to four degenerate bases (Ns) to improve sequence diversity. PCRs were prepared in a total volume of 20 μL for each sample containing 10 μL MyFi Mix (Meridian Bioscience), 0.16 μL of Bovine Serum Albumin (20 mg/mL, Thermo Scientific), 5.84 μL of UltraPure Distilled Water (Invitrogen), 1 μL of each forward and reverse primer (10 μM, Eurofins) and 2 μL of template DNA. We ran all PCRs in triplicate under the following thermocycling conditions: 95°C for 10 min, followed by 40 cycles of 95°C for 30 s, 60°C for 45 s and 72°C for 30 s, and a final elongation of 72°C for 5 min for Tele02 12S primers and polymerase activation at 95°C for 10 min, followed by 35 cycles of 94°C for 1 min, 45°C for 1 min, 72°C for 1 min and a final elongation of 72°C for 5 min for COI primers. Replicates were pooled, and samples were visualised on a 2% agarose gel stained with SYBRsafe (Invitrogen) to check for the successful amplification of target fragments. PCR products were then purified with Mag‐Bind TotalPure NGS magnetic beads (Omega Bio‐tek Inc), adding to 30 μL of PCR products a 1× ratio and a 0.8× ratio of magnetic beads for 12S and COI, respectively (Bronner et al. 2009). Purified DNA was quantified using a Qubit Flex 4.0 fluorometer with the Qubit dsDNA HS Assay Kit (Invitrogen). Based on the total DNA concentration, samples were normalised and pooled in equimolar concentration. The four libraries (i.e., two libraries for Tele02 12S, one for eDNA filter and metaprobe COI and one for cobble COI) were prepared using the NEXTFLEX Rapid DNA‐Seq Kit 2.0 for Illumina platforms (PerkinElmer) according to the manufacturer's protocol. The Agilent 4200 TapeStation and High Sensitivity D1000 ScreenTape (Agilent Technologies) indicated secondary products (e.g., adaptor dimers) remained, which were removed by an additional magnetic bead clean‐up (1× ratio for all the libraries). Libraries were quantified using quantitative PCR (qPCR) on a Rotor‐Gene Q (Qiagen) with the NEBNext Library Quant Kit for Illumina (New England Biolabs). We diluted Tele02 12S libraries to 1 nM and COI libraries to 4 nM; all final libraries and PhiX Control were re‐quantified using qPCR before sequencing. Tele02 12S libraries were sequenced separately at 85 pM with 20% PhiX Control on an Illumina iSeq 100 using the i1 Reagent v2 (300‐cycle; Illumina Inc.). The two COI libraries were pooled in equimolar concentration and sequenced together at 12.5 pM with 10% PhiX Control using V3 chemistry (2 × 250 bp paired‐end) on an Illumina MiSeq platform.

### 2.3 Bioinformatic Processing

Bioinformatic procedures were based on the OBITOOLS software 1.2.11 (Boyer et al. 2016). We first assessed the quality of reads (R1 and R2) with FASTQC and then trimmed low quality ends using OBICUT with a minimum lenght threshold of 150 bp for 12S and 240 bp for COI. Paired‐end reads were aligned by ILLUMINAPAIREDEND with a quality score > 40. We used NGSFILTER to demultiplex samples based on their unique barcodes, allowing for a single base mismatch. Sequences were filtered via OBIGREP to remove singletons and retain the expected read length range (129–209 bp for 12S; 300–325 bp for COI), and dereplicated via OBIUNIQ. We removed chimeras with UCHIME (Edgar et al. 2011) and clustered the remaining sequences into Molecular Operational Taxonomical Units (MOTUs) with SWARM (Mahé et al. 2015) setting the threshold to *d* = 3 for Tele02 12S and *d* = 13 for COI (Wangensteen et al. 2018). Taxonomic assignment for each MOTU of Tele02 12S data was performed with (1) SINTAX (Edgar 2016) and BLASTn (v2.11.0) using the FishCARD curated Californian fish reference database (Gold et al. 2021) and (2) ECOTAG algorithm using a 12S database created through an in silico PCR on the whole EMBL database (Release version r143) implemented with ECOPCR (a reference database of 26,387 sequences was obtained). The final taxonomic assignment of each MOTU was determined by seeking a consensus among the three assignment methods. First, MOTUs for which all three methods provided the same taxonomic classification were assigned accordingly. When consensus among all three methods was not possible, MOTUs that were assigned to the same taxon by both SINTAX and BLAST with the FishCARD curated Californian fish reference database were assigned based on this agreement. Finally, remaining MOTUs were assigned according to ECOTAG results. For COI, we performed taxonomic assignment of each MOTU using the ECOTAG algorithm with a custom‐made metazoan database of 279,692 sequences created through an in silico PCR against the EMBL database (r143) implemented with ECOPCR. Taxonomic assignment was then validated using BLASTn. Both 12S and COI datasets were filtered, removing potential contamination using the control samples (blanks and negative controls) with the DECONTAM package in R (Davis et al. 2018), using the prevalence method and a threshold of 0.5. To reduce the effects of low‐abundance false positives due to tag switching and/or cross‐contamination, only reads with an occurrence > 10 were retained (Schnell et al. 2015). Refinement of the 12S datasets consisted of removing: (1) non‐marine taxa (e.g., bovine and human), (2) taxa that could not unambiguously be assigned to the genus or species rank, and (3) MOTUs showing < 95% identity. For COI data, non‐target MOTUs (i.e., terrestrial taxa) and MOTUs assigned to prokaryotes and fungi were removed. Two COI datasets were then generated: (i) a filtered taxonomic dataset where all the unclassified and < 80% identity MOTUs were removed and (ii) a MOTU dataset including also poorly classified and unclassified MOTUs. All downstream statistical analyses of COI were performed on both the taxonomically assigned and the MOTU datasets in order to examine the influence of taxonomic assignment and filtering on the results. Finally, the following species were excluded from final datasets: positive controls (i.e., *Pangasianodon hypophthalmus* for Tele02 12S and *Penaeus vannamei* for COI) and two mainly tropical genera (i.e., *Amphiprion* and *Thalassoma*) identified by Tele02 12S FRAM samples whose detection was not expected along the Californian coast and may probably originate from background environmental contamination.

### 2.4 Data Analyses

Statistical analyses were performed separately for the two markers as the two target fragments were sequenced with different sequencing depths and targeted a very distinct range of taxa (i.e., Tele02 12S to specifically amplify teleosts while COI all metazoans). Boxplots were used to compare the performance of sampling methods (i.e., BAM, DAM, FRAM and water for 12S, and BAM, cobbles, DAM and water for COI) in terms of taxon richness. The significance of comparisons was assessed by Kruskal–Wallis test and Mann–Whitney *U*‐test for unpaired data, adjusting *p*‐values for multiple testing (Bonferroni correction). In order to assess whether each of the eDNA sampling methods fully captured the species diversity, we examined relationships between species richness (number of identified taxa) and sampling effort (number of samples), calculating accumulation curves for each sampling method separately with the iNEXT R‐package (Hsieh et al. 2016). iNEXT provides functions to compute and plot rarefaction and extrapolation curves together. Based on the total number of taxa estimated by the accumulation curve and the slope of the curve, the number of samples required to capture 80% and 90% of the expected diversity was calculated by dividing the cumulative number of expected taxa by the estimated total number of taxa for each marker and each sampling method separately. For COI data, because of the high proportion of MOTUs removed by taxonomic assignment and filtering procedures (see Tables S2 and S4 for details), boxplots and accumulation curves were performed both on the filtered taxonomic dataset and on the MOTU dataset including also poorly classified and unclassified MOTUs. To explore possible differences between sampling methods in their ability to detect marine taxa across multiple phyla (using just COI data), the proportion of MOTUs and reads per phylum by each sampling method separately was calculated and visualised through bar plots. For the more common phyla (i.e., Amoebozoa, Anellida, Arthropoda, Bacillariophyta, Bryozoa, Chlorophyta, Chordata, Cnidaria, Dinoflagellata, Echinodermata, Mollusca, Ochrophyta, Porifera, and Rhodophyta), we further examined the percentage of total MOTUs per phylum detected by each of the four sampling methods through a Circos plot (Krzywinski et al. 2009). Venn diagrams were built, using the VENNDIAGRAM package in R (Chen and Boutros 2011), to compare the overall taxa detection among BAM, DAM and water for 12S, and BAM, cobbles, DAM and water for COI. For 12S species, we also calculated and visualised the proportion of pelagic over demersal taxa (Froese and Pauly 2022) in DAM and water samples, as we expected demersal fishes to be preferentially detected by DAM samples because of the divers' proximity to the seafloor. We explored the vertebrate community composition (beta‐diversity) across sampling sites using square‐root transformed reads datasets for all the semi‐quantitative analyses. A bubble plot was drawn to visualise the vertebrate relative proportional read counts per each sampling site and a non‐metric multidimensional scaling (nMDS) was performed with the ‘metaMDS’ function in the R package VEGAN (Oksanen et al. 2018) based on Bray‐Curtis distance on a dataset including all taxa detected by the two 12S libraries together. We excluded FRAM samples because they were collected from a different habitat and the low number of species detected compared to all other sampling methods could artificially affect beta‐diversity (see results). Polygons were generated on the nMDS ordination to represent the two biogeographic areas, north and south of *Humqaq*. Differences among sampling sites and biogeographic areas were then assessed via a PERMANOVA test using the ‘adonis’ function in the R‐package VEGAN with 9999 permutations. We further investigated differences in community assemblages across sampling sites considering the whole metazoan community (COI dataset) through nMDS. COI data‐based nMDS were based on Jaccard distance matrices calculated on both general MOTU and taxonomically refined datasets and were performed considering all the sampling methods (i.e., BAM, cobbles, DAM and water) or only the methods that were carried out north and south of *Humqaq* (i.e., BAM and water), together or separately. COI community analyses were performed using binary presence/absence data because cobble and aqueous eDNA samples were extracted with different methods and this could bias the proportionality of the reads in the output (Hallmaier‐Wacker et al. 2018). Differences among sampling sites, sampling methods and sampling areas were assessed via a PERMANOVA test using the ‘adonis’ function with 9999 permutations. For 12S and COI data separately, we finally identified taxa associated with statistically significant differences among sampling areas (north and south *Humqaq*) using an indicator species analysis with the ‘multipatt’ function in the INDICSPECIES R‐package (De Cáceres and Legendre 2009).

All the described analyses were performed in R v4.3.6.3 (R Core Team 2023).

## 3 Results

### 3.1 Sequencing Depth

High throughput sequencing resulted in a total of 3,321,705 and 2,966,016 raw reads for the 48 and 39 samples respectively in the two 12S libraries; 8,923,199 raw reads for the 72 samples in the COI library from water and metaprobes; 5,285,343 raw reads for the 80 samples in the cobble COI library. Following bioinformatic analyses, taxonomic assignment, data filtering, and contaminant removal, the two 12S datasets consisted of 749,462 reads (median per sample = 12,293) and 679,167 reads (median per sample = 15,532). After combining them, a total of 52 taxa (42 teleosts, one elasmobranch, three birds, four mammals and two echinoderms) were retained for ecological analyses (Table S6). Following removal of contaminants and low abundance, four samples were excluded for downstream analysis (see Table S3 for details). We obtained 2,280,187 reads (median per sample = 45,390) for the eDNA water‐and‐metaprobe COI MOTU dataset, and 1,875,104 reads (median per sample = 9571) for the cobble COI MOTU dataset, resulting in a total of 2086 and 3069 COI MOTUs respectively. 1,651,464 reads (median per sample = 28,385) and 247 taxa were retained in the water‐and‐metaprobes taxonomically filtered dataset, and 1,177,714 reads (median per sample = 7603) and 374 taxa in the cobble taxonomically filtered dataset (see Tables S2 and S4).

The number of reads per sample in the COI final datasets varied widely. As expected, the number of taxa seen in a sample increased with read depth, with an asymptote for samples above 10,000. Samples with reads numbering above 10,000 showed a variable number of taxa, but taxon number was not correlated with reads (Figure S1). To minimise the potential influence of sequencing depth on taxa richness and strengthen the statistical power of method comparisons, we restricted our statistical analysis to COI samples with read depth above 10,000. After this exclusion criterion, the final taxonomic/MOTU COI datasets consisted of eight/nine BAM samples (424,399/607,954 reads), nine/nine DAM samples (367,027/483,366 reads), 15/17 water samples (769,266/1,172,120 reads) and 27/28 cobble samples (1,116,533/1,790,706 reads), respectively. In the 12S final dataset, only one sample (one of the two replicates of Shearwater1 water sample) had less than 1000 reads. We removed this sample and restricted statistical analysis to samples with reads > 1000 to limit correlation between read depth and number of detected species (Figure S1). The final 12S dataset included: six BAM samples (90,779 reads), eight DAM samples (123,070 reads), seven FRAM samples (259,821 reads) and 17 water samples (744,613 reads).

### 3.2 Sampling Methods Comparison

**FIGURE 2:**
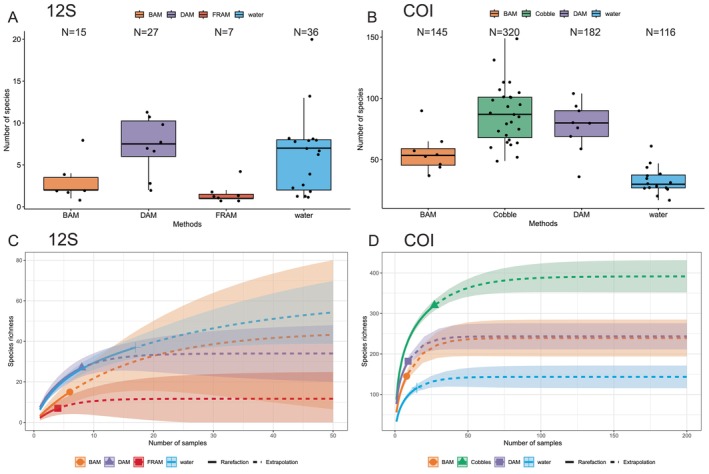
Comparison of the sampling methods performance in terms of taxon detection. Boxplots represent the number of species per sample detected by Tele02 12S primers (A) and universal COI primers (B). ‘*N*’ indicates the total number of taxa identified by each method separately. Rarefaction curves depict the total taxa detected in the collected samples by Tele02 12S (C) and COI (D). Curves are split based on the sampling method (i.e., BAM, DAM, FRAM and water for 12S; BAM, cobbles, DAM and water for COI).

For 12S data, water and DAM samples enabled the detection of significantly more species (mean 6.2 and 7.4, respectively) than BAM and FRAM (3 and 1.6, respectively; Kruskal–Wallis: *H* = 12.58, df = 3, *p* = 0.005), with no significant differences between water and DAM samples (Mann–Whitney test: *U* = 78.5, *p* = 0.39; Figure 2A).

For COI data, cobble samples returned a remarkably higher total number of taxa compared to water eDNA and metaprobe methods (i.e., 320 for cobbles, 145 for BAM, 182 for DAM and 116 for water). However, considering the mean number of species per sample, DAM and cobble samples had a similar mean number of species (76.5 and 87.5 taxa respectively), significantly higher compared to BAM (56 taxa) and water samples (33 taxa; Figure 2B; Kruskal–Wallis: *H* = 35.9, df = 3, *p* < 0.001), while the differences among them were not statistically significant (Mann–Whitney test: *U* = 150, *p* = 0.31). These differences were also observed when considering all the MOTUs (Figure S2A): the mean number of MOTUs per sample was also considerably higher in DAM and cobbles samples compared to the other methods (Kruskal–Wallis: *H* = 33, df = 3, *p* < 0.001). For COI data, ‘traditional’ sampling from surface waters yielded a significantly lower mean number of taxa per sample compared to all the other methods (Mann–Whitney test: *U* = 662, *p* < 0.001).

Species accumulation curves attested that, for both markers and across methods, the number of samples was not sufficient to capture the whole taxon diversity across the study area (Figure 2C,D); the analysis of taxon richness for 12S revealed that between 35% and 79% of the estimated total species were detected. Similar to the analysis of taxon numbers, DAM and water samples showed the highest fraction of 12S biodiversity. For COI, between 61% and 82% of the estimated total COI taxa and between 55% and 70% of the estimated total COI MOTUs were detected (Table 1). Accumulation curves indicated that the BAM method would require a significant increase in sampling effort (i.e., 18 and 22 additional samples for 12S and COI respectively) to reach 80% of the estimated diversity. All methods would still necessitate a substantial addition of samples (i.e., BAM 28, DAM 11, FRAM 11 and water 19 for 12S; BAM 49, DAM 37, water 35 and cobbles 34 for COI) to attain 90% of the expected diversity (Figures 2C,D and S2B; Table 1).

**TABLE 1:** Number of samples per each method required to detect 80% and 90% of the total taxa richness, respectively, estimated from the rarefaction curves.

Method	*n*	% obs diversity	*n* samples needed to reach % estimated diversity	
			80%	90%
12S				
BAM	6	35%	24	34
DAM	8	79%	11	19
FRAM	7	58%	11	18
Water	17	70%	24	36
COI				
BAM	8	61%	30	57
DAM	9	75%	20	46
Water	15	81%	15	50
Cobbles	27	82%	27	61
COI MOTUs				
BAM	9	55%	37	65
DAM	9	63%	31	58
Water	17	70%	38	67
Cobbles	28	55%	75	117

*Note:* ‘*n*’ is the number of samples analysed and ‘% obs diversity’ is the percentage of the total estimated diversity observed per each method.

**FIGURE 3:**
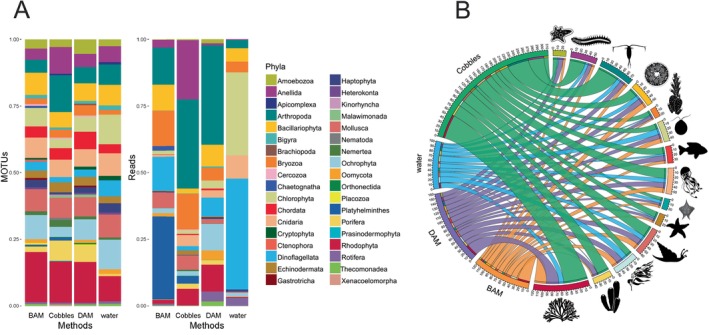
Bar plots showing the relative proportion of MOTUs (left) and reads (right) per phylum as returned by COI data for each of the four sampling methods separately (A). The Circos Plot represents the percentage of total MOTUs per phylum detected by each of the four sampling methods, considering only the 14 most abundant phyla (B). From top to bottom: Amoebozoa, Annelida, Arthropoda, Bacillariophyta, Bryozoa, Chlorophyta, Chordata, Cnidaria, Dinoflagellata, Echinodermata, Mollusca, Ochrophyta, Porifera and Rhodophyta.

The proportion of MOTUs per phylum identified by COI was similar among the four sampling methods (i.e., BAM, cobbles, DAM and water), with negligible differences between them (Figure 3A; Table S5). However, when considering read proportions, cobble samples had considerably higher proportions of typically benthic phyla (e.g., Annelida, Bryozoa, Rhodophyta and Arthropoda of the orders Isopoda and Amphipoda), while water samples were dominated by pelagic taxa (e.g., Chlorophyta, Cnidaria and Dinoflagellata; Figure 3A). Interestingly, in the reads returned by DAM samples, the highest proportions belonged to a mixture of benthic and pelagic phyla (e.g., Bacillariophyta, Dinoflagellata, Ochrophyta, Rhodophyta and Arthropoda including both pelagic copepods and benthic isopods and amphipods). Considering only the most abundant phyla, we were able to document differences also in the percentage of total MOTUs per phylum identified by each of the four sampling methods, with a considerable prevalence of cobble MOTUs from typically benthic phyla (e.g., Annelida, Porifera, Rhodophyta and Arthropoda over‐represented by isopods and amphipods; Figure 3B).

**FIGURE 4:**
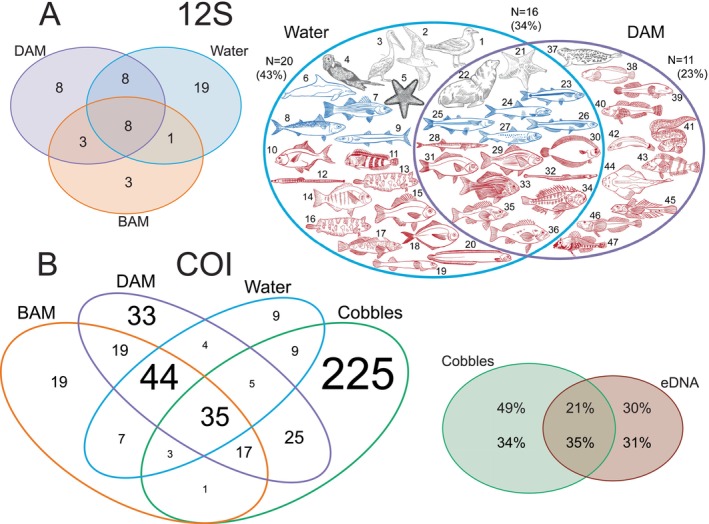
Venn Diagrams of the taxa detected by (A) Tele02 12S and (B) universal COI. For 12S, the species identified by BAM, DAM and water are compared in the left diagram while the species detected only by DAM and water are illustrated on the right. In grey are non‐marine‐fish taxa, in blue pelagic taxa and in red demersal taxa. See Table S6 for the names of taxa denoted by numbers. For COI, the four sampling methods are shown separately in the left graph, with font size proportional to the number of taxa; eDNA methods (i.e., BAM, DAM and water) appear combined in the right diagram. In this last diagram, the percentages above correspond to the percentage of species in each area, while those below to the percentage of reads belonging to the taxa in each area.

Among the 52 total taxa revealed by 12S eDNA metabarcoding, two fish (i.e., *Oligocottus snyderi* , *Menticirrhus undulatus* ) were exclusively returned by FRAM samples (Table S6), whereas two species were also seen in all other sample types and four species were also seen in water samples. Three species were exclusive to BAM, eight to DAM and 19 to water samples (Figure 4A). Eight species were shared between BAM, DAM and water. When comparing only the species returned by DAM and water (the samples with the highest species number), 34% of taxa were detected by both sampling methods. Of these taxa, the percentage of demersal species was 56% in shared species and 55% in exclusively water samples (Figure 4A). By strong contrast, 100% of fish species only detected with the DAM method were demersal species.

Of the 455 total taxa returned by COI, 95 were identified by metaprobes, ‘traditional’ water filtering and cobble samples. By contrast, 225 were exclusively returned by cobble and 135 exclusively by metaprobe or water samples (Figure 4B).

### 3.3 Community Structure and Ecological Patterns

**FIGURE 5:**
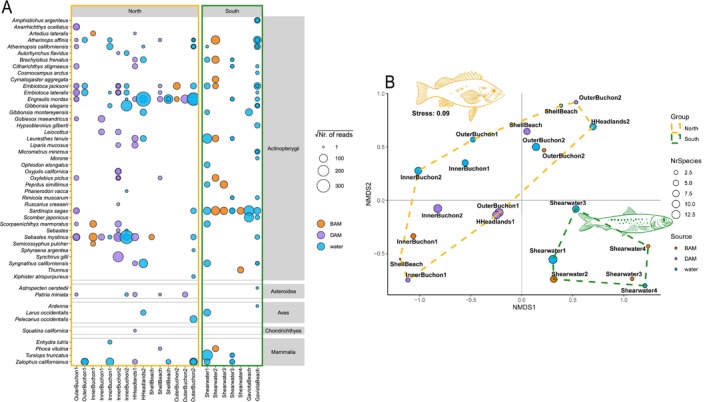
Bubble plot representing the vertebrate relative abundance (transformed number of reads) per each sampling site as returned by Tele02 12S data (A). Pattern of the species assemblages across sampling sites (considering only the offshore sites), as returned by the non‐metric multidimensional scaling (nMDS) with Bray‐Curtis distance and based on 12S eDNA data (B). Points represent samples coloured according to the sampling methods. Polygons separate samples according to the biogeographic area, north and south *Humqaq*. Silhouettes depict the top contributing species to each group differentiation, according to the indicator species analysis (i.e., *Sebastes mystinus* for the north and *Sardinops sagax* for the south).

For 12S data, both the bubble plot (Figure 5A) and the nMDS (Figure 5B) showed a clear community structure with significant influence of the geographic area, north and south of *Humqaq*: polygons on the nMDS plot emphasised the separation of these two areas along the first axis (PERMANOVA: *R* ^2^ = 0.17, *p* < 0.001). The indicator species analysis revealed three species as characteristic of the regions because of their relatively higher read abundance: the blue rockfish ( *Sebastes mystinus* ) in the north, and the Pacific sardine ( *Sardinops sagax* ) and the California grunion ( *Leuresthes tenuis* ) in the south (Table S7). These distinctions were apparent across sampling types, excluding FRAM samples.

**FIGURE 6:**
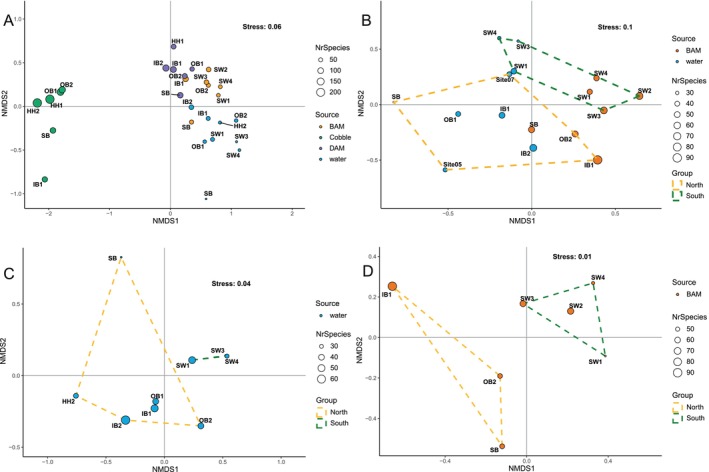
Pattern of community composition across sampling sites as returned by the non‐metric multidimensional scaling (nMDS) with Jaccard distance and based on presence/absence taxonomic COI data. The ordination plots are based on (A) all COI data, (B) BAM and water, (C) only water and (D) only BAM samples. Points represent samples and are coloured according to the sampling methods (i.e., BAM, cobbles, DAM and water). Polygons on graphs B, C and D separate samples according to the biogeographic area, north and south of *Humqaq*.

nMDS analysis of BAM and water COI samples, which were the only ones collected both south and north of *Humqaq* for COI, also showed clear separation between the two geographic areas (Figures 6B–D and S4B–D).

The nMDS‐based assemblage structure resulting from COI data considering all samples showed a significant influence of sampling method on species assemblage patterns. The separation of the four methods (i.e., BAM, cobbles, DAM and water) was evident in the ordination plots in Figure 6A and was supported by PERMANOVA results (*R* ^2^ = 0.43, *p* < 0.001). This separation was further highlighted when the ordination plot was generated considering all the MOTUs (Figure S4A—*R* ^2^ = 0.26, *p* < 0.001 for the MOTU‐based dataset). The plot in Figure 6B, which represented BAM and water samples together, emphasised a north–south distinction in both datasets (along the y‐axis), while also showing that the methods differed in species composition (along the x‐axis; PERMANOVA: *R* ^2^ = 0.18, *p* < 0.001 for the methods; *R* ^2^ = 0.15, *p* = 0.002 for the area). Indicator species analysis for water and BAM samples identified five taxa strongly associated with the north (i.e., the rotifer *Synchaeta*, the green alga *Chloropicon sieburthii*, the chrysophyte alga *Dinobryon*, clams of the genus *Pitar* and the dinoflagellate *Dinophysis*) and four with the south (i.e., the gastrotrich *Urodasys*, the flagellate *Chattonella* and the algae *Ostreococcus*, and *Phaeocystis globosa* ; Table S7). Lack of cobble samples from south of *Humqaq* precluded a similar analysis for this dataset.

## 4 Discussion

Our study tested a variety of DNA‐based sampling approaches to document diversity and spatial patterns of kelp forest species along the US‐California coast in the newly designated Chumash Heritage National Marine Sanctuary. Samples included encrusted cobbles, standard water filtering and various deployments of artificial passive water samplers (metaprobes). These methods provided different but complementary views of kelp forest biodiversity from fish to invertebrates to algae, providing comparative ecological data on over 400 species. Such an effort epitomises the increasing efforts being made worldwide to develop efficient and robust monitoring tools to better characterise biodiversity patterns and chart the distribution of species across the globe. DNA metabarcoding, with its rapid, accurate and universal way of collecting multitaxon information, is expected to become a mainstay in routine biomonitoring programs in every habitat and across all domains of the tree of life (Pawlowski et al. 2021). However, the collection of environmental samples for later sequence‐based biodiversity screening requires consideration of various practical factors, including efficiency, resources, scalability and sustainability, to name a few (Bessey et al. 2021; Hansen et al. 2020; Jeunen et al. 2022; Maiello et al. 2023).

### 4.1 Kelp Forests Diversity

The heterogeneity of sampling methods together with the moderate number of samples in this study underscores the need for a larger number of replicates/samples, which is especially important in hyper‐diverse ecosystems for the detection of rarer taxa and in field studies lacking ground‐truthing data (Ficetola et al. 2015). However, using a combination of relatively low‐cost/effort sampling techniques, we identified over 400 marine species/genera: 64 chordates, 58 crustaceans, 20 echinoderms, 42 molluscs, 42 annelids, 35 cnidarians, 31 sponges, 77 red algae, 22 brown algae, 26 diatoms, and 10 dinoflagellates. Importantly, it should be noted that DNA sequencing revealed, perhaps unsurprisingly, a large fraction of unclassified marine biodiversity (see Tables S2 and S4 for details on the number of reads and MOTUs removed from the taxonomically refined dataset). This ‘biodiversity gap’ is particularly remarkable for cobble samples; 35% of cobble MOTUs were unclassified, compared to only 12% unclassified COI MOTUs from water filter‐and‐metaprobe samples. Poor taxonomic description and low representation in DNA reference databases of many benthic invertebrate species that live and hide in and on cobble rocks are two likely explanations (Derycke et al. 2010; Shum et al. 2019).

Among the 52 vertebrate species detected by the 12S marker, there were some iconic species of kelp forest ecosystems, such as rockfishes (*Sebastes* spp.), Señorita wrasse ( *Oxyjulis californica* ), lingcod ( *Ophiodon elongatus* ), cabezon ( *Scorpaenichthys marmoratus* ) and surfperches ( *Embiotoca jacksoni* and *E. lateralis* ), all highly important for fisheries and ecosystem health in the region, and whose molecular signal has previously been found on coastal Californian kelp ecosystems (Lamy et al. 2021; Monuki et al. 2021; Port et al. 2016). Interestingly, eDNA also identified more elusive and cryptic species, such as wolfeel ( *Anarrhichthys ocellatus* ), tidepool sculpin (*Oligocottus maculatus*) and black prickleback ( *Xiphister atropurpureus* ), rarely spotted by visual census and often under‐represented in surveys (Port et al. 2016). Despite the Tele02 primers being designed to specifically target teleosts, they are known to regularly amplify also the DNA of other vertebrate taxonomic groups present in the environment, such as elasmobranchs, mammals and birds, which all represent additional valuable biodiversity information in monitoring contexts (Mariani, Harper, et al. 2021; Ritter et al. 2022). Here, 12S metabarcoding data returned also two sea stars ( *Patiria miniata* and *Astropecten oerstedii*), the near‐threatened angel shark ( *Squatina californica* ), seabirds (*Ardenna* shearwaters; Western gull, *Larus occidentalis* ; Brown pelican, *Pelecanus occidentalis* ), and marine mammals (Harbour Seal, *Phoca vitulina* ; Common bottlenose dolphin, *Tursiops truncatus* ; Californian sea lion, *Zalophus californicus*; Sea Otter, *Enhydra lutris* ), all of which are well‐known marine species of Californian coastal environments, some (e.g., *Squatina californica, Pelecanus occidentalis* and *Zalophus californicus*) with a very important ecological role as top predators of kelp forest ecosystems (Foster and Schiel 2010).

Similarly, cobble samples returned many of the species expected by visual censusing to be present including giant kelp ( *Macrocystis pyrifera* ) and the purple sea urchin ( *Strongylocentrotus purpuratus* ), two important components in kelp ecosystem patterns. However, in addition there were many understory species that seldom occur in wide‐scale ecological assessments of kelp forest communities because of their small size or the difficulty of recognising them in the field. The ornate tubeworm *Diopatra ornata* , the small‐bladed red alga *Isabbottia ovalifolia* and the encrusting sponge *Halichondria panicea* all occurred in over 90% of our samples but are rare from most kelp forest survey data.

### 4.2 Sampling Methods Comparison

Cobble samples returned a substantially higher number of both taxa and MOTUs compared to aqueous environmental DNA samples: 374 and 247 taxa and 3069 and 2086 MOTUs respectively (Figures 2D and S2B). There are two possible explanations for this observed pattern. The first one is related to differences in the sequencing effort: for most of the sampling sites where cobbles were collected (with the exception of Inner Buchon2), the number of cobble replicates analysed exceeded the ones for each eDNA method. The second reason is intrinsically linked with the methodology: most of the material metabarcoded from cobble samples belongs to organisms encrusting to substrate, while aqueous eDNA consists of traces of DNA dispersed in the water, where the DNA is considerably less concentrated and more degraded. Furthermore, whilst eDNA from water contains a well‐mixed amount of a myriad of DNA templates, the diverse ‘community DNA’ on cobbles depends on the specific micro‐community settled on a given rock and therefore exhibits a greater degree of inter‐sample variation. In the comparison between cobble and aqueous eDNA samples, it is important to consider that a substantial proportion of cobble samples (~60%) were excluded from the analysis due to the chosen read depth threshold (< 10,000 reads), with subsequent implications on the power of cobble samples to retrieve biodiversity information. This highlights the importance of future method optimization and potential increase of replication and/or sequencing depth to improve the quality of metabarcoding data. Interestingly, the mean number of taxa/MOTUs per sample were very similar between cobble and diver‐metaprobe samples, while ‘traditional’ sampling from surface waters yielded a significantly lower number of taxa (Figures 2B and S2A). Previous studies found strong eDNA signal stratification on the water column (Andruszkiewicz et al. 2017; Jeunen et al. 2020) and Monuki et al. (2021) recovered considerably higher diversity at depths compared to surface water along the coast of California, arguing that this might be related to both chemical (e.g., UV irradiation and temperature) and more biological (e.g., DNA and other pelagic particles sinking at major depths) processes. Given the limited bathymetric distance here, the lower diversity returned by COI surface water data compared to the more bentho‐demersal cobble and DAM samples might be further explained by the action of divers stirring the substrate while collecting cobble rocks, which likely resuspends sedimented DNA. When we compared the taxa returned by cobbles and eDNA (considering metaprobes and water eDNA together), only ~20% of species were detected by both methods, but they included the most common and abundant organisms: 35% of the total COI reads belonged to these shared taxa. Among the four tested eDNA sampling methods (i.e., BAM, DAM, FRAM and water), boat‐assisted metaprobes (BAM) and metaprobes cast from the shore (FRAM) typically returned a lower number of species. Of the eight fish species identified by FRAM samples, two were exclusive of this sampling method: the fluffy sculpin ( *Oligocottus snyderi* ), often found in rocky intertidal and subtidal areas, and the California corbina ( *Menticirrhus undulatus* ), which is typically associated with coastal sandy environments. The different results obtained with FRAM samples are partly due to the different habitat explored, but in general the low diversity in BAM and FRAM samples warrant the need for further testing and optimisation of both the boat‐assisted and fishing rod‐assisted techniques. The versatility, affordability, and opportunistic nature of these variations of metaprobe use make it worth further methodological refinement, potentially including an increase of soaking time, which was only ~15 min compared to the 30/40 min of DAM samples. Additional movement of the metaprobes through the water column might also enhance the absorption of DNA particles dispersed in the ocean by the gauze rolls.

Diver‐assisted metaprobes (DAM) and filtered water bottles were the most efficient in terms of vertebrate species identification (Figure 2A). Many of the 16 (representing the 34%) taxa shared between the two methods (Figure 4A) were characteristic species usually very abundant in the investigated area, such as blue rockfish ( *Sebastes mystinus* ) and black and striped surfperches (*Embitoca jacksoni* and *E. lateralis* ) and included commercially important Californian fishes, such as the northern anchovy ( *Engraulis mordax* ), the Pacific sardine ( *Sardinops sagax* ) and the speckled sanddab ( *Citharichthys stigmaeus* ). DAM samples typically identified more cryptic benthic taxa (e.g., *Anarrhichthys ocellatus* , *Oxylebius pictus* , *Synchirus gilli* , and *Squatina californica* ), while water samples retrieved a higher proportion of pelagic fishes and marine birds, in closer contact with the surface of the ocean (Figure 4A). DAM samples were collected while divers were collecting and handling cobbles, in proximity to where demersal fishes feed and hide, compared to the upper layers of the water column, where the water samples were gathered. Despite the type of fish community captured by DAM and water samples proving to be essentially the same, the diver activity near the bottom allowed the detection of more cryptic, less mobile species, while still capturing the most abundant nektonic, schooling species, which tends to be over‐represented in aqueous eDNA samples (Aglieri et al. 2021).

This ‘demersal dividend’ yielded by the DAM method during cobble collections was also corroborated by COI results, which returned a very similar pattern of different proportional detection of benthic/planktonic taxa across the whole metazoan community (Figure 3). The most abundant phyla (in terms of proportion of reads) in cobble samples were benthic phyla that live on the seabed like annelids (Syllidae, Sabellariidae, Polynoidae and Terebellidae), crustaceans (Amphipoda and Isopoda), bryozoans, molluscs, sponges and red algae (Rhodophyta). In kelp forest ecosystems, the incrusting red coralline algae that cover many cobbles are crucial for the post‐larval settlement of many kelp‐associated animals (O'Leary et al. 2017). Previous work on cobble eDNA from Monterey, CA showed a large number of species on each sampled cobble (Shum et al. 2019) and enabled comparisons of population genetic differences among conspecifics on different cobbles (Shum and Palumbi 2021). These snapshots of diversity on each cobble allowed examination of diversity and variance in cobble ecological communities over large and small scales. They also provide an assessment of a wide range of small species that play an important role in the bottom of the kelp forest food chain. In water samples, instead, planktonic phyla such as diatoms (Bacillariophyta), green algae (Chlorophyta), cnidarians and dinoflagellates prevailed. Studies have previously shown that eDNA metabarcoding of filtered seawater is unable to provide a complete picture of the epibenthic community, only returning a small fraction of it even when using universal primers (Hajibabaei et al. 2019; Antich et al. 2021). Interestingly, the highest percentage of COI reads in DAM samples belonged to a mixture of the demersal/benthic component as isopods and amphipods, brown seaweed (Ochrophyta), red algae and the pelagic taxa mainly represented by copepods, diatoms and dinoflagellates (Figure 3). Diver‐assisted metaprobes might thus represent a particularly versatile eDNA sampling approach (exemplified in Figure 4B), which can capture several of the benthic organisms typically encrusting cobbles while also detecting high proportions of the planktonic organisms usually abundant in marine eDNA samples. The metaprobe thus represents an agile, reusable and inexpensive eDNA sampling tool, which is considerably less labour‐intensive than both water filtering and cobble collection. Unsurprisingly, DAM data miss several taxa that can be detected with more traditional methods (as cobbles and water filtration), but it appears to be a good compromise for gathering species inventories from both the pelagic and the bentho/demersal realms. The potential integration of metaprobes with diver activity in routine monitoring programmes offers several advantages. The DAM is a cost‐effective sampling approach that can easily be standardised and implemented across various locations, providing high‐throughput data that can yield valuable ecological insights into diverse components of marine ecosystems, particularly when screened with multiple markers. Similarly, deploying metaprobes from shore by fishing rods is a simple way of sampling a wide range of shorelines. The low yield of species in the current dataset from FRAM samples calls for further development of this approach.

### 4.3 Community Structure and Ecological Patterns

Despite differences among the tested sampling methods, DNA metabarcoding samples produced rich species inventories that can serve as baselines for future, regular monitoring of these areas. Irrespective of sampling method or genetic marker used, community structure was deeply influenced by the biogeographic break around *Humqaq*. Vertebrate beta‐diversity clearly separated the two regions north and south of *Humqaq* (Figure 5). We detected this separation using square‐root transformed reads as a rough proxy of abundance, but similar patterns were also found using presence/absence data (Figure S3). Recent studies have suggested the possibility of using read depth in eDNA metabarcoding data (Clark et al. 2020; Guri et al. 2023; Mariani, Fernandez, et al. 2021), where a proxy for species abundance could help delineate species assemblage differences. This approach may be easiest to apply for water‐based sampling, where larger fragments of organisms are less common. Since read depth is notoriously sensitive to body size, sampling and amplification artefacts, read abundance values are often transformed to reduce these issues. A presence‐absence approach can avoid many of these problems but requires large numbers of samples to create a robust dataset (Shum et al. 2019).

Changes in composition and abundance across the *Humqaq* break could be explicitly tracked for individual species, in some cases paralleling existing knowledge of these species along the central Californian coast. *Sebastes mystinus* is a very common and abundant fish species in nearshore habitats off northern California and is found to be vastly more abundant north of the break (e.g., see figure 9 in Sakuma et al. 2006). By contrast, in the southern area we found a high abundance of the California grunion, *Leuresthes tenuis* , which has a distribution ranging from Monterey Bay to Baja California but is much more uncommon north of *Humqaq* (Fritzsche et al. 1985). We could further observe opposite trends in two main small pelagic fishes in coastal California, with the northern anchovy ( *Engraulis mordax* ) much more abundant in the North, and the Pacific sardine ( *Sardinops sagax* ) dominant in the South (Figure 5A). These two species, which together play a key role in sustaining the pelagic food web along the western North America coast, are very mobile and migratory species (Weber and McClatchie 2010), with known long‐term, quasi‐decadal variability in abundance (Reiss et al. 2008). However, little is known about their fine‐scale spatial and temporal separation along the Californian coast. It is reasonable to expect that further eDNA surveys over multiple seasons and years may resolve the phenology of these key ecological players. Their co‐occurrence with likely prey species and predators (Djurhuus et al. 2020; D'Alessandro and Mariani 2021) may help develop a framework to characterise trophic links and monitor possible changes in ecosystem structure.

The detection of the iconic California sheephead ( *Semicossyphus pulcher* ) only at one sampling site inside the marine protected area of Point Buchon was unexpected because the species is usually not common north of *Humqaq* (Ziegler et al. 2023). However, the species can be carried north by warm water currents during heatwaves (Cornish and Dormeier 2010), which occurred strongly between 2015 and 2016, prior to our sampling in 2022. In fact, California sheephead is now seen as far north as Monterey Bay. California sheephead has long been central to fisheries management strategies due to its vulnerability to exploitation (Alonzo et al. 2004); yet, because of its sensitivity to large‐scale sea surface warming patterns and its role in sea urchin population regulation, it may also represent one of the several species whose fluctuations may be of great value to monitor broader ecosystem changes.

Although we lacked cobble and DAM samples for the sites south of *Humqaq*, due to unfavourable weather conditions, we found COI‐based evidence of the biogeographic boundary in the pelagic samples (Figures 6B–D and S4B–D). Forthcoming work will include underwater sampling across the whole study area, and in additional seasons, which will allow a more comprehensive bentho‐pelagic characterisation of community change and a more in‐depth analysis of inter‐specific ecological links. Interestingly, indicator species analysis on pelagic samples revealed the over‐representation in the area south of *Humqaq* of two algal taxa (i.e., *Chattonella* and *Phaeocystis globosa* ) that are known to cause harmful algal blooms. Red tides off the coast of Southern California have captured the attention of scientists and the public over the last decades (McGowan et al. 2017), as their toxicity triggers mass animal mortalities (Curtiss et al. 2008; Lewitus et al. 2012) and major damages to ecosystem services. Both *Chattonella* sp. and *Phaeocystis globosa* have previously been regarded as harmful algal species (Núñez‐Vázquez et al. 2011; Wang et al. 2021), so their detection in the study area offers an additional example of the potential benefits of the application of DNA‐based methods for whole biodiversity screening, assisting with the early detection of pernicious taxa that can have destructive ecosystem‐wide impacts (Liu et al. 2020).

## 5 Conclusion

Despite uncertainties around eDNA particle transport through prevailing currents (Andruszkiewicz et al. 2019), multiple studies have already reported spatial variation at a very small spatial scale (Yamamoto et al. 2016; Port et al. 2016), even in very dynamic, exposed coastal ecosystems, such as the kelp forests along the California coast (Monuki et al. 2021). The suite of sampling tools examined here demonstrated the power of eDNA analysis to serve as a multi‐purpose asset for the monitoring of rare and iconic species, the identification of spatial breaks, the portrayal of complex benthic and pelagic communities, and the characterisation of harmful algae. Although no single eDNA method can guarantee a complete suite of data to fully address all of the above priorities, there is sufficient evidence to indicate that eDNA metabarcoding is mature for implementation as a dynamic, reproducible approach to monitor faunal changes in kelp habitats along the California coast. Where scuba activities are possible, diver‐assisted metaprobes—screened at both COI and 12S markers—may represent the most comprehensive single approach to characterise across benthic, planktonic and nektonic communities. When surveys are carried out from the boat, standard water‐filtering methods would be most appropriate. With additional multi‐season investigations forthcoming across the whole study area, we expect that more data will strengthen the efficiency of our metabarcoding‐based methods for biodiversity monitoring, with the view to test their effectiveness in other regions and habitats.

## Author Contributions

G.M., M.R.L., S.R.P. and S.M. conceptualised the study and designed the methodology. G.M., M.R.L. and E.A.H. collected samples. G.M. and M.R.L. conducted all laboratory work. G.M. and E.F.N. carried out bioinformatic and statistical analyses. G.M. drafted the manuscript with major contribution from S.R.P. and S.M. All authors provided feedback on the manuscript and gave final approval for publication.

## Conflicts of Interest

The authors declare no conflicts of interest.

## Supporting information


Data S1.

## Data Availability

The raw sequence data files, metadata, MOTU tables and final datasets can be accessed at https://doi.org/10.5061/dryad.02v6wwqfv. Bioinformatic pipelines and R scripts used for statistical analysis and to generate figures are publicly available from https://github.com/GiuliaMaiello/Multi‐tool‐marine‐metabarcoding‐in‐kelp‐habitat.
