# Supplementary material for: Multi‐Tool Marine Metabarcoding Bioassessment for Baselining and Monitoring Species and Communities in Kelp Habitats
Source: Mol Ecol Resour. 2025 Jul 17;25(7):e70010. doi: 10.1111/1755-0998.70010 (PMC12415811; doi:10.1111/1755-0998.70010)
Supplement: Supplementary file 1 — Data S1. [file MEN-25-e70010-s001.pdf]

MOLECULAR ECOLOGY RESOURCES

Supplemental Information for:

Multi-tool marine metabarcoding bioassessment for baselining and monitoring species and communities in kelp habitats

Giulia Maiello[1], Marilla R. Lippert[2], Erika F. Neave[1,3], Erik A. Hanson[2], Stephen R. Palumbi[2*] & Stefano Mariani[1*]

[1]School of Biological and Environmental Sciences, Liverpool John Moores University, Liverpool, United Kingdom.

[2]Hopkins Marine Station, Department of Biology, Stanford University, Pacific Grove, CA, USA

[3]Department of Life Sciences, Natural History Museum, London, United Kingdom.

[*] These authors equally contributed to this work

Corresponding author:

Giulia Maiello, School of Biological and Environmental Sciences, Liverpool John Moores University, Byrom Street, L3 3AF, Liverpool, UK. Email: G.Maiello@ljmu.ac.uk

Table of Contents:

Supplementary Table S1	Page 2
Supplementary Table S2	Page 3
Supplementary Table S3	Page 4
Supplementary Table S4	Page 6
Supplementary Table S5	Page 10
Supplementary Table S6	Page 12
Supplementary Table S7	Page 14
Supplementary Figure S1	Page 15
Supplementary Figure S2	Page 16
Supplementary Figure S3	Page 17
Supplementary Figure S4	Page 18

MOLECULAR ECOLOGY RESOURCES

Table S1 – Name, date, geographical coordinates (datum = WGS84), location and type of collected samples (B = BAM, C = cobbles, D = DAM, W = water, F = FRAM) are given for each sampling site.

Site	Date	Lat (°N)	Long (°W)	Location	Sample type
**Outer Buchon 1 (OB1)**	22/09/2022	35.18	120.81	offshore	CDW
**Inner Buchon 1 (IB1)**	22/09/2022	35.24	120.89	offshore	BCDW
**Inner Buchon 2(IB2)**	22/09/2022	35.25	120.9	offshore	CDW
**Harmony Headlands 1 (HH1)**	23/09/2022	35.48	121.03	offshore	CDW
**Harmony Headlands 2 (HH2)**	23/09/2022	35.46	121.01	offshore	BCDW
**Shell Beach (SB)**	25/09/2022	35.15	120.69	offshore	BDW
**Outer Buchon 2 (OB2)**	25/09/2022	35.17	120.79	offshore	BCDW
**Shearwater 1 (SW1)**	29/09/2022	34.54	120.55	offshore	BW
**Shearwater 2 (SW2)**	29/09/2022	34.45	120.44	offshore	BW
**Shearwater 3 (SW3)**	29/09/2022	34.45	120.38	offshore	BW
**Shearwater 4 (SW4)**	29/09/2022	34.45	120.36	offshore	BW
**Bluff Trail (BT)**	22/09/2022	35.27	120.89	shore	F
**Mussel Point (MP)**	22/09/2022	34.93	120.66	shore	F
**Surf Beach (SurfB)**	23/09/2022	34.68	120.61	shore	F
**Shell Beach (SB)**	25/09/2022	35.15	120.65	shore	F
**Gaviota Beach (GB)**	28/09/2022	34.47	120.23	shore	FW

MOLECULAR ECOLOGY RESOURCES

Table S2 – Total number of reads at different step of the bioinformatic process are given for each primer set (Tele02 and COI) and each library separately.

Tele02

		Library 1	Library 2
**Total raw reads**		3,321,705	2,966,016
**After bioinformatic analysis**	Reads	2,274,420	2,106,255
	MOTUs	393	295
**Assigned taxonomy (Metazoa)**	Reads	2,213,166	2,065,618
	MOTUs	286	193
**Unassigned**	Reads	61,254	40,637
	MOTUs	107	102
**Assigned to target taxa**	Reads	1,123,857	1,059,762
	MOTUs	207	115
**Assigned to non-target taxa**	Reads	1,089,308	1,005,856
	MOTUs	79	83
**Unique target taxa after filtering**	Reads	749,462	679,167
	Taxa	36	30

COI

		Metaprobe&eDNA	Cobbles
**Total raw reads**		8,923,199	5,285,343
**After bioinformatic analysis**	Reads	2,394,402	2,688,931
	MOTUs	2,845	12,906
**Assigned taxonomy (Eukaryota)**	Reads	2,312,999	2,578,054
	MOTUs	2,523	9,685
**Unassigned**	Reads	81,403	110,877
	MOTUs	322	3,221
**Assigned to target taxa**	Reads	1,760,058	1,974,503
	MOTUs	2,320	8,992
**Assigned to non-target taxa**	Reads	553,941	603,551
	MOTUs	203	693
**Unique MOTU dataset**	Reads	2,280,187	1,875,104
	MOTUs	2,086	3,069
**Unique taxa assigned dataset after filtering**	Reads	1,651,464	1,177,714
	Taxa	247	374

MOLECULAR ECOLOGY
RESOURCES

Table S3 – Total number of reads and MOTUs/taxa returned by Tele02 12S primers are given for each sampling site separately at different filtering steps: (i) the raw MOTU table, (ii) the MOTU table after removal of contaminants (iii) the final filtered taxonomic dataset (where <95% taxa were removed). For each sample, the collection method and the geographic location are given. Samples highlighted in bold represent the ones that were kept for downstream analysis (> 1,000 reads).

			**Raw MOTU table**		**Non contaminant MOTU table**		**Final taxonomic dataset**	
**Sample**	**Source**	**Geo**	**Nr Reads**	**Nr MOTUs**	**Nr Reads**	**Nr MOTUs**	**Nr Reads**	**Nr Taxa**
**OB1**	**DAM**	**North**	**131084**	**57**	**9702**	**30**	**9690**	**16**
**OB1**	**DAM**	**North**	**84311**	**86**	**15260**	**28**	**15248**	**16**
**OB1**	**water**	**North**	**12051**	**18**	**11409**	**10**	**11409**	**9**
**OB1**	**water**	**North**	**5561**	**11**	**5244**	**5**	**5244**	**5**
**IB1**	**BAM**	**North**	**46573**	**23**	**14962**	**11**	**14957**	**7**
**IB1**	BAM	North	6495	3	1	2	1	2
**IB1**	**DAM**	**North**	**68757**	**31**	**8162**	**9**	**8162**	**9**
**IB1**	DAM	North	15209	5	3	3	3	3
**IB1**	**water**	**North**	**47391**	**27**	**12297**	**18**	**12294**	**12**
**IB1**	water	North	4805	5	3	3	3	3
**IB2**	**DAM**	**North**	**36043**	**43**	**10673**	**24**	**10669**	**16**
**IB2**	**DAM**	**North**	**67195**	**48**	**49940**	**25**	**50025**	**15**
**IB2**	**water**	**North**	**81382**	**31**	**47488**	**22**	**47501**	**17**
**IB2**	**water**	**North**	**106519**	**14**	**106274**	**10**	**106273**	**7**
**HH1**	**DAM**	**North**	**103684**	**59**	**15540**	**20**	**15540**	**17**
**HH1**	DAM	North	45497	5	4	4	4	4
**HH1**	water	North	5	5	4	4	4	4
**HH2**	BAM	North	64700	10	7	6	7	6
**HH2**	DAM	North	0	0	0	0	0	0
**HH2**	**DAM**	**North**	**3999**	**10**	**3999**	**10**	**1414**	**6**
**HH2**	**water**	**North**	**8813**	**2**	**8813**	**2**	**8813**	**2**
**HH2**	**water**	**North**	**196514**	**20**	**181859**	**16**	**181857**	**15**
**SB**	**BAM**	**North**	**42729**	**10**	**1738**	**7**	**1738**	**7**
**SB**	**BAM**	**North**	**38714**	**14**	**38159**	**11**	**38158**	**7**
**SB**	DAM	North	14274	6	3	3	3	3
**SB**	**DAM**	**North**	**55255**	**68**	**1717**	**15**	**1717**	**12**
**SB**	**FRAM**	**North**	**121935**	**26**	**16124**	**9**	**16122**	**8**
**SB**	FRAM	North	35847	17	247	11	247	11
**SB**	**FRAM**	**North**	**1588**	**5**	**1586**	**4**	**1586**	**3**
**SB**	**water**	**North**	**49351**	**13**	**16435**	**9**	**16435**	**8**

**SB**	**water**	**North**	**17049**	**26**	**8893**	**8**	**8893**	**6**
**OB2**	**BAM**	**North**	**32131**	**18**	**5080**	**9**	**5080**	**8**
**OB2**	BAM	North	115060	27	27	10	27	8
**OB2**	**DAM**	**North**	**29256**	**8**	**12531**	**6**	**12529**	**5**
**OB2**	DAM	North	39355	6	8	5	8	5
**OB2**	**water**	**North**	**86696**	**22**	**82327**	**19**	**82327**	**12**
**OB2**	**water**	**North**	**95520**	**18**	**95501**	**16**	**95501**	**14**
**SW1**	**BAM**	**South**	**97454**	**11**	**38822**	**7**	**38822**	**6**
**SW1**	BAM	South	7	5	5	3	5	3
**SW1**	**water**	**South**	**36857**	**13**	**11241**	**6**	**11241**	**6**
**SW1**	**water**	**South**	**96065**	**32**	**88209**	**26**	**88202**	**19**
**SW2**	**BAM**	**South**	**66411**	**23**	**48931**	**16**	**48931**	**11**
**SW2**	water	South	14	6	2	3	2	3
**SW3**	**water**	**South**	**18491**	**10**	**13609**	**6**	**13609**	**6**
**SW3**	BAM	South	7	4	3	3	3	3
**SW3**	**water**	**South**	**2707**	**21**	**2351**	**12**	**2351**	**12**
**SW3**	**water**	**South**	**4347**	**18**	**3803**	**12**	**3803**	**12**
**SW4**	**BAM**	**South**	**11354**	**8**	**7012**	**4**	**7012**	**4**
**SW4**	BAM	South	22221	3	0	0	0	0
**SW4**	water	South	31287	10	2	2	2	2
**SW4**	**water**	**South**	**64191**	**16**	**55730**	**10**	**55730**	**9**
**GB**	**FRAM**	**Coast**	**6744**	**3**	**1815**	**2**	**1815**	**2**
**GB**	**FRAM**	**Coast**	**46315**	**12**	**38495**	**10**	**38495**	**7**
**GB**	**water**	**Coast**	**102548**	**127**	**27397**	**111**	**27184**	**29**
**GB**	**water**	**Coast**	**12447**	**36**	**1509**	**25**	**1506**	**13**
**BT**	**FRAM**	**Coast**	**50518**	**16**	**28789**	**12**	**28788**	**10**
**BT**	FRAM	Coast	35925	9	5	4	5	4
**BT**	**FRAM**	**Coast**	**111187**	**18**	**104073**	**16**	**104072**	**10**
**MP**	**FRAM**	**Coast**	**30068**	**7**	**30065**	**5**	**30065**	**5**
**MP**	**FRAM**	**Coast**	**86469**	**18**	**72423**	**12**	**72423**	**10**
**MP**	**FRAM**	**Coast**	**92876**	**12**	**92858**	**10**	**92854**	**7**
**SurfB**	**FRAM**	**Coast**	**86537**	**47**	**4695**	**7**	**4695**	**7**
**SurfB**	**FRAM**	**Coast**	**39477**	**17**	**22777**	**15**	**22775**	**12**

MOLECULAR ECOLOGY
RESOURCES

Table S4 – Total number of reads and MOTUs/taxa returned by COI primers are given for each sampling site separately at different filtering steps: (i) the raw MOTU table, (ii) the MOTU table where we removed contaminants but we kept poorly assigned and unassigned MOTUs and (iii) the filtered taxonomic dataset where <80% and unclassified MOTUs were removed. For each sample, the collection method and the geographic location are given. Samples highlighted in bold represent the ones that were kept for statistical analysis (> 10,000 reads).

			Raw MOTU table		MOTU dataset		Taxonomic dataset	
**Site**	**Source**	**Geo**	**NrReads**	**NrSp**	**NrReads**	**NrTaxa**	**NrReads**	**NrTaxa**
**OB1**	Cobble	North	132	13	132	13	87	4
**OB1**	**Cobble**	**North**	**82453**	**442**	**45757**	**363**	**30134**	**84**
**OB1**	Cobble	North	1041	29	1001	28	568	5
**OB1**	Cobble	North	1731	38	944	29	808	4
**OB1**	Cobble	North	608	47	371	43	52	2
**OB1**	**Cobble**	**North**	**55219**	**1704**	**54333**	**1643**	**22863**	**154**
**OB1**	Cobble	North	172	15	8	3	3	1
**OB1**	Cobble	North	442	37	147	22	44	4
**OB1**	**Cobble**	**North**	**14108**	**512**	**12723**	**482**	7255	93
**OB1**	Cobble	North	5122	357	2268	300	1406	60
**OB1**	**DAM**	**North**	**34671**	**845**	**34321**	**830**	**26833**	**98**
**OB1**	DAM	North	50	1	50	1	0	0
**OB1**	**water**	**North**	**73975**	**513**	**73911**	**505**	**47988**	**62**
**OB1**	**water**	**North**	**46571**	**341**	**46512**	**334**	**31606**	**49**
**IB1**	Cobble	North	1530	14	5	4	50	2
**IB1**	**Cobble**	**North**	**109886**	**1012**	**109670**	**986**	**88888**	**132**
**IB1**	Cobble	North	2931	28	300	18	219	9
**IB1**	Cobble	North	10512	269	10299	252	8047	67
**IB1**	Cobble	North	1064	45	182	34	83	9
**IB1**	Cobble	North	746	38	202	26	47	5
**IB1**	Cobble	North	497	100	417	90	208	27
**IB1**	Cobble	North	3110	61	2989	46	2908	9
**IB1**	Cobble	North	12820	200	5025	146	2644	41
**IB1**	Cobble	North	5848	116	1872	78	1054	28
**IB1**	**BAM**	**North**	**48483**	**768**	**48051**	**751**	**30944**	**120**
**IB1**	BAM	North	522	13	520	12	342	7

**IB1**	**DAM**	**North**	**81322**	**516**	**80912**	**503**	**67090**	**92**
**IB1**	**DAM**	**North**	**72083**	**941**	**71178**	**920**	**56088**	**117**
**IB1**	**water**	**North**	**96610**	**507**	**96531**	**494**	**77573**	**79**
**IB1**	**water**	**North**	**53257**	**79**	**53217**	**75**	**38426**	**24**
**IB2**	Cobble	North	241	50	102	38	24	8
**IB2**	Cobble	North	92	3	33	1	33	1
**IB2**	Cobble	North	77	6	2	2	0	0
**IB2**	Cobble	North	176	19	24	15	0	0
**IB2**	Cobble	North	691	61	247	38	165	11
**IB2**	Cobble	North	288	38	128	25	95	9
**IB2**	Cobble	North	1467	69	891	46	528	18
**IB2**	Cobble	North	5889	171	1792	113	905	34
**IB2**	Cobble	North	12920	224	9741	193	5005	36
**IB2**	Cobble	North	11309	165	7164	124	4385	39
**IB2**	**DAM**	**North**	**35935**	**948**	**34914**	**833**	**24268**	**123**
**IB2**	**DAM**	**North**	**51938**	**1046**	**50892**	**1020**	**30583**	**131**
**IB2**	**water**	**North**	**93350**	**643**	**93229**	**632**	**74811**	**98**
**IB2**	**water**	**North**	**57000**	**64**	**56988**	**61**	**54406**	**29**
**HH1**	**Cobble**	**North**	**63096**	**2011**	**62341**	**1928**	**20146**	**180**
**HH1**	**Cobble**	**North**	**61914**	**2806**	**60793**	**2709**	**26619**	**155**
**HH1**	**Cobble**	**North**	**33987**	**1596**	**31987**	**1490**	**17438**	**126**
**HH1**	**Cobble**	**North**	**54750**	**2168**	**53048**	**2056**	**37477**	**178**
**HH1**	**Cobble**	**North**	**47096**	**1547**	**32407**	**1462**	**19633**	**131**
**HH1**	**Cobble**	**North**	**103815**	**828**	**44694**	**795**	**39504**	**97**
**HH1**	**Cobble**	**North**	**47160**	**2029**	**33848**	**1981**	**18747**	**126**
**HH1**	**Cobble**	**North**	**76935**	**2967**	**75517**	**2882**	**38708**	**190**
**HH1**	**Cobble**	**North**	**63774**	**1796**	**62343**	**1708**	**53052**	**136**
**HH1**	**Cobble**	**North**	**197576**	**4298**	**185895**	**4169**	**55086**	**210**
**HH1**	BAM	North	103	8	68	3	66	1
**HH1**	**DAM**	**North**	**47657**	**863**	**47075**	**846**	**36975**	**109**
**HH1**	DAM	North	3	2	3	2	0	0
**HH1**	water	North	303	8	303	8	251	1
**HH1**	water	North	350	7	350	7	236	4
**HH2**	**Cobble**	**North**	**79358**	**2630**	**78815**	**2543**	**60584**	**160**

HH2	Cobble	North	33214	1210	31946	1175	17528	120
HH2	Cobble	North	85681	1726	71306	1675	48860	154
HH2	Cobble	North	109384	3163	105477	3068	54411	192
HH2	Cobble	North	88066	2012	84007	1948	56752	131
HH2	Cobble	North	91264	1528	56802	1479	45406	131
HH2	Cobble	North	87650	2460	84916	2380	55106	156
HH2	Cobble	North	37889	1658	37051	1620	22109	133
HH2	Cobble	North	133374	4042	125346	3910	53356	205
HH2	Cobble	North	72428	2696	68210	2622	41543	177
HH2	BAM	North	420	12	362	7	223	2
HH2	BAM	North	78	3	78	3	78	3
HH2	DAM	North	348	9	348	9	322	5
HH2	DAM	North	45	2	45	2	0	0
HH2	water	North	150885	143	150879	140	145767	35
HH2	water	North	120190	182	120163	177	117065	47
SB	Cobble	North	27112	108	2716	75	1361	17
SB	Cobble	North	9988	246	3246	178	1899	45
SB	Cobble	North	79301	1344	78938	1299	61160	115
SB	Cobble	North	6943	180	3784	127	1983	35
SB	Cobble	North	2383	109	801	77	411	25
SB	Cobble	North	20570	86	9621	60	437	16
SB	Cobble	North	2128	42	430	28	348	13
SB	Cobble	North	11714	125	977	86	661	34
SB	Cobble	North	75094	268	73424	240	38459	67
SB	Cobble	North	8293	201	5805	166	3320	52
SB	BAM	North	64844	380	64544	329	57564	62
SB	BAM	North	6	1	0	0	0	0
SB	DAM	North	60247	160	59873	158	57294	37
SB	DAM	North	81341	1021	77686	998	42522	116
SB	water	North	27110	137	27037	130	17394	37
SB	water	North	17128	231	17115	226	7753	31
OB2	Cobble	North	108965	1205	105689	1164	82802	135
OB2	Cobble	North	1591	159	1540	137	662	31
OB2	Cobble	North	1984	141	1002	112	463	36

**OB2**	Cobble	North	3512	136	2683	127	1845	30
**OB2**	Cobble	North	3908	144	1627	126	182	10
**OB2**	Cobble	North	1661	135	1277	116	304	21
**OB2**	**Cobble**	**North**	**28235**	**1124**	**26969**	**1043**	**16907**	**144**
**OB2**	Cobble	North	3271	123	374	73	186	26
**OB2**	Cobble	North	4336	73	2767	58	1428	22
**OB2**	Cobble	North	10009	160	6444	124	3338	39
**OB2**	**BAM**	**North**	**68654**	**382**	**68299**	**362**	**55565**	**65**
**OB2**	**BAM**	**North**	**12267**	**74**	**12068**	**71**	8806	24
**OB2**	**DAM**	**North**	**40272**	**485**	**39271**	**476**	**26508**	**83**
**OB2**	DAM	North	113	7	113	7	49	2
**OB2**	**water**	**North**	**84658**	**562**	**84380**	**548**	**26217**	**58**
**OB2**	**water**	**North**	**58049**	**429**	**57581**	**422**	**29497**	**56**
**SW1**	BAM	South	0	0	0	0	0	0
**SW1**	**BAM**	**South**	**77531**	**224**	**77193**	**218**	**60880**	**48**
**SW1**	**water**	**South**	**44137**	**420**	**44114**	**411**	**18302**	**57**
**SW1**	**water**	**South**	**82287**	**580**	**82244**	**573**	**55027**	**83**
**SW2**	BAM	South	99	3	99	3	71	2
**SW2**	**BAM**	**South**	**66995**	**660**	**66898**	**650**	**40640**	**90**
**SW2**	water	South	264	6	264	6	63	1
**SW2**	water	South	1254	36	1240	35	250	8
**SW3**	**BAM**	**South**	**75541**	**645**	**75392**	**634**	**22143**	**80**
**SW3**	**BAM**	**South**	**76236**	**218**	**76194**	**214**	**50674**	**45**
**SW3**	**water**	**South**	**38309**	**401**	**38270**	**394**	9567	48
**SW3**	**water**	**South**	**54137**	**446**	**54038**	**438**	**12656**	**45**
**SW4**	**BAM**	**South**	**126184**	**389**	**126118**	**382**	**106659**	**65**
**SW4**	BAM	South	28	3	6	2	0	0
**SW4**	water	South	7446	57	7446	57	1748	8
**SW4**	**water**	**South**	**88400**	**479**	**88370**	**471**	**23822**	**53**

MOLECULAR ECOLOGY RESOURCES

Table S5 – List of all the phyla detected by COI DNA metabarcoding. The number of MOTUs and the number of reads detected by each sampling method (i.e., BAM, cobbles, DAM and water) are given per each phylum.

	BAM		Cobbles		DAM		Water	
	Nr MOTUs	Nr reads	Nr MOTUs	Nr reads	Nr MOTUs	Nr reads	Nr MOTUs	Nr reads
Amoebozoa	5	961	10	3426	10	5531	3	111
Anellida	6	12558	35	256541	9	2891	7	1736
Apicomplexa	0	0	2	110	0	0	1	32
Arthropoda	7	60190	48	385274	11	137409	9	24606
Bacillariophyta	12	41925	20	21701	12	29884	9	39585
Bigyra	2	391	3	1658	2	1871	2	80
Brachiopoda	0	0	0	0	1	15	0	0
Bryozoa	3	57314	10	153119	7	17671	2	31856
Cercozoa	1	12	2	4460	1	119	0	0
Chaetognatha	1	7495	0	0	0	0	0	0
Chlorophyta	10	5857	12	15795	10	10951	13	245761
Chordata	6	633	15	4440	12	2542	4	361
Cnidaria	11	3359	23	48637	12	9838	10	68299
Cryptophyta	1	30	0	0	2	60	2	134
Ctenophora	1	82	0	0	0	0	0	0
Dinoflagellata	5	55497	7	18079	5	26315	7	328771
Echinodermata	4	790	13	19225	6	998	3	240
Gastrotricha	1	174	0	0	1	563	0	0
Haptophyta	4	1442	2	366	4	1696	4	6237
Heterokonta	1	92	1	2759	1	169	1	13
Kinorhyncha	0	0	1	345	0	0	0	0
Malawimonada	0	0	3	441	2	39	0	0
Mollusca	12	25513	26	59629	13	6063	10	3067
Nematoda	0	0	3	734	0	0	0	0
Nemertea	2	83	9	5996	2	382	1	24
Ochrophyta	13	8203	13	19828	14	36854	13	11647
Oomycota	2	5395	0	0	2	12989	2	1090
Orthonectida	0	0	1	2440	1	140	0	0
Placozoa	1	11	0	0	1	95	0	0
Platyhelminthes	1	134460	4	31700	0	0	0	0
Porifera	3	507	26	22014	11	6475	1	31
Prasinodermophyta	0	0	1	216	0	0	0	0
Rhodophyta	27	6355	54	71589	28	37112	11	1865

**Rotifera**	1	2947	2	1019	1	13699	1	24174
**Thecomonadea**	1	636	1	1000	1	6064	1	27
**Xenacoelomorpha**	0	0	1	12	0	0	0	0

Table S6 – List of all the taxa detected by Tele02 12S DNA metabarcoding of BAM, DAM, FRAM and water samples separately. The number column (Nr) refers to the numbers in Figure 4A.

Nr	Scientific name	Class	BAM	DAM	FRAM	Water
14	*Amphistichus argenteus*	Actinopterygii			X	X
41	*Anarrhichthys ocellatus*	Actinopterygii		X		
2	*Ardenna*	Aves				X
40	*Artedius lateralis*	Actinopterygii	X	X		
5	*Astropecten oerstedii*	Asteroidea				X
23	*Atherinops affinis*	Actinopterygii	X	X		X
25	*Atherinopsis californiensis*	Actinopterygii		X		X
28	*Aulorhynchus flavidus*	Actinopterygii		X		X
29	*Brachyistius frenatus*	Actinopterygii	X	X		X
30	*Citharichthys stigmaeus*	Actinopterygii		X		X
12	*Cosmocampus arctus*	Actinopterygii				X
	Cymatogaster aggregata	Actinopterygii	X			
31	*Embiotoca jacksoni*	Actinopterygii	X	X		X
33	*Embiotoca lateralis*	Actinopterygii		X		X
24	*Engraulis mordax*	Actinopterygii	X	X	X	X
4	*Enhydra lutris*	Mammalia				X
13	*Gibbonsia elegans*	Actinopterygii			X	X
16	*Gibbonsia montereyensis*	Actinopterygii				X
39	*Gobiesox maeandricus*	Actinopterygii		X		
11	*Hypsoblennius gilberti*	Actinopterygii				X
1	*Larus occidentalis*	Aves			X	X
46	*Leiocottus*	Actinopterygii		X		
26	*Leuresthes tenuis*	Actinopterygii	X	X	X	X
38	*Liparis mucosus*	Actinopterygii		X		
	Menticirrhus undulatus	Actinopterygii			X	
15	*Micrometrus minimus*	Actinopterygii				X
7	*Morone*	Actinopterygii				X
	Oligocottus snyderi	Actinopterygii			X	
10	*Ophiodon elongatus*	Actinopterygii				X
42	*Oxyjulis californica*	Actinopterygii		X		

MOLECULAR ECOLOGY
RESOURCES

**43**	*Oxylebius pictus*	Actinopterygii	X	X		
**21**	*Patiria miniata*	Asteroidea		X		X
**3**	*Pelecanus occidentalis*	Aves				X
**18**	*Peprilus simillimus*	Actinopterygii	X			X
**10**	*Phanerodon vacca*	Actinopterygii				X
**37**	*Phoca vitulina*	Mammalia	X	X		
**19**	*Rimicola muscarum*	Actinopterygii				X
**47**	*Ruscarius creaseri*	Actinopterygii		X		
**27**	*Sardinops sagax*	Actinopterygii	X	X		X
**8**	*Scomber japonicus*	Actinopterygii				X
**34**	*Scorpaenichthys marmoratus*	Actinopterygii	X	X		X
**35**	*Sebastes*	Actinopterygii		X		X
**36**	*Sebastes mystinus*	Actinopterygii	X	X		X
	Semicossyphus pulcher	Actinopterygii	X			
**9**	*Sphyraena argentea*	Actinopterygii				X
**44**	*Squatina californica*	Chondrichthyes		X		
**32**	*Syngnathus californiensis*	Actinopterygii		X		X
**45**	*Synchirus gilli*	Actinopterygii		X		
	Thunnus	Actinopterygii	X			
**6**	*Tursiops truncatus*	Mammalia				X
**20**	*Xiphister atropurpureus*	Actinopterygii				X
**22**	*Zalophus californianus*	Mammalia		X		X

MOLECULAR ECOLOGY RESOURCES

Table S7 - Species identified by the indicator species analyses as significantly characteristic (significance at α= 0.05) of each region (i.e., North and South *Humqaq*) are given for each primer set (i.e., 12S and COI) separately. Results are sorted by relative influence from top to down

			Stat	p-value
**12S**				
	**North**	*Sebastes mystinus*	0.775	0.035
	**South**	*Sardinops sagax*	0.98	< 0.001
		Leuresthes tenuis	0.66	0.05
**COI**				
	**North**	*Synchaeta*	0.943	0.0012
		Dinobryon	0.88	0.003
		Dinophysis	0.81	0.01
		Pitar	0.82	0.01
		Chloropicon sieburthii	0.81	0.04
	**South**	*Chattonella*	0.84	0.006
		Phaeocystis globosa	0.82	0.01
		Ostreococcus	0.756	0.04
		Urodasys	0.718	0.01

MOLECULAR ECOLOGY RESOURCES

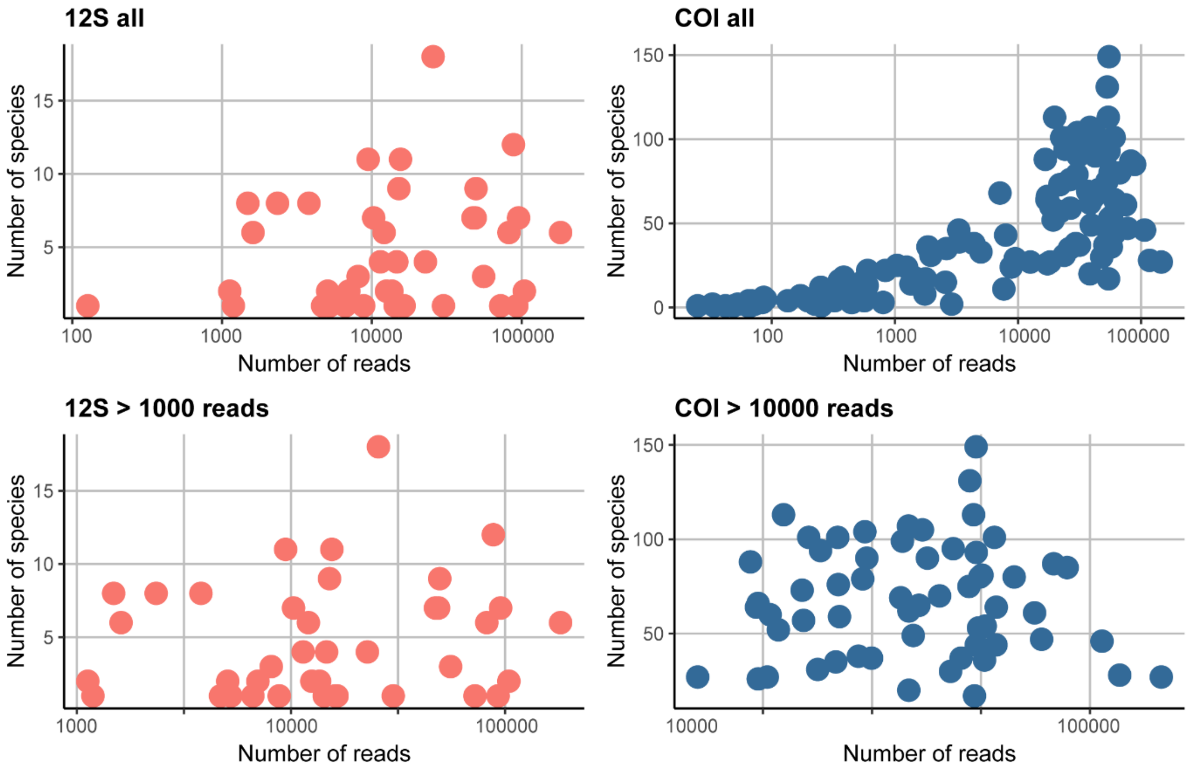

Figure S1 – Scatterplots representing the relationship between the number of reads (logarithmic scale) and the number of identified species. The two plots on the top include all the samples, while the plots on the bottom only samples with read depth below 1,000 for 12S and below 10,000 for COI.

MOLECULAR ECOLOGY RESOURCES

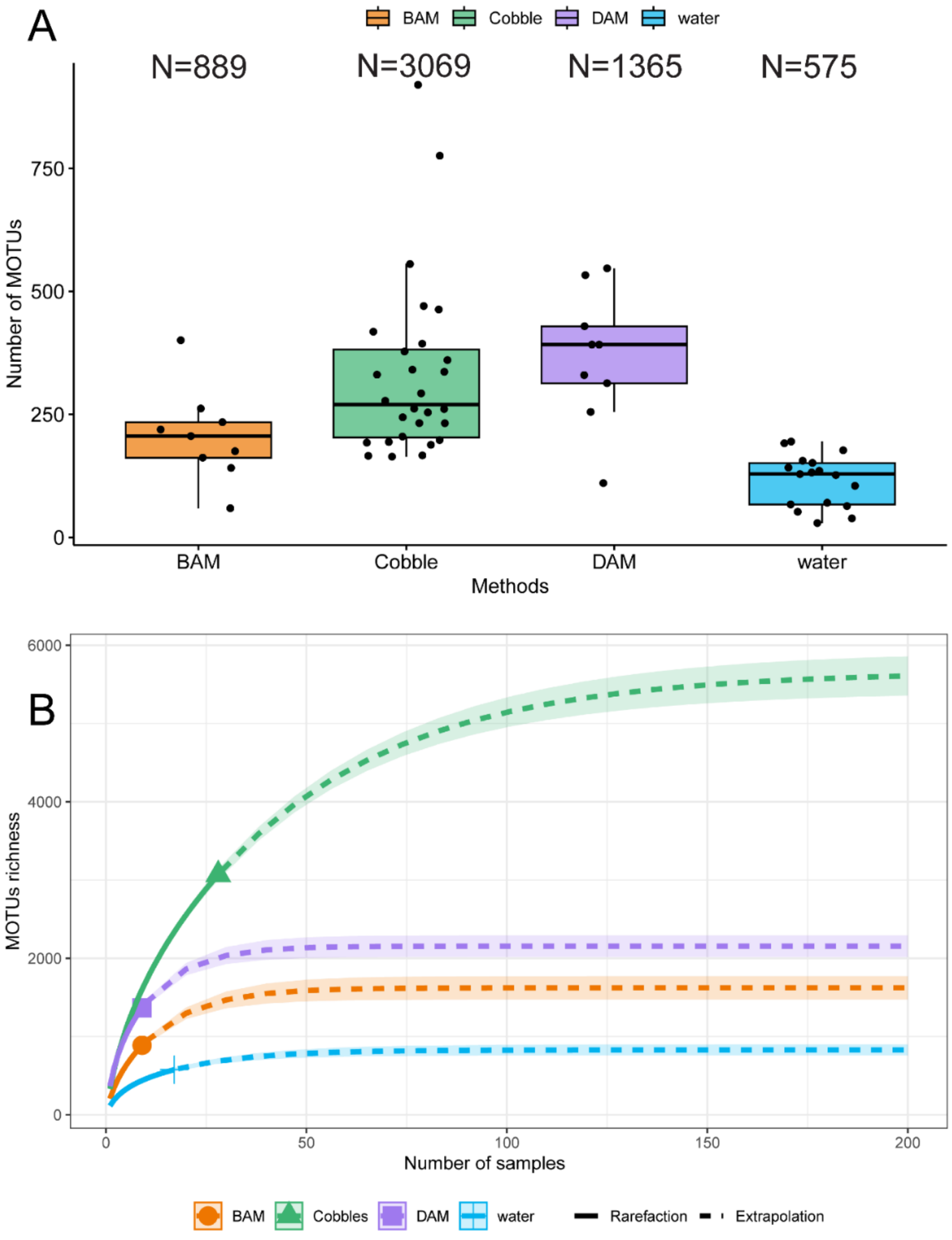

Figure S2 – Comparison of the sampling methods performance in terms of MOTU detection. Boxplots represent the number of MOTU per samples detected by COI primers (A). Rarefaction curves depict the total MOTUs detected in the collected samples by COI primers (B). Curves are split based on the sampling method (i.e., BAM, cobbles, DAM and water).

MOLECULAR ECOLOGY
RESOURCES

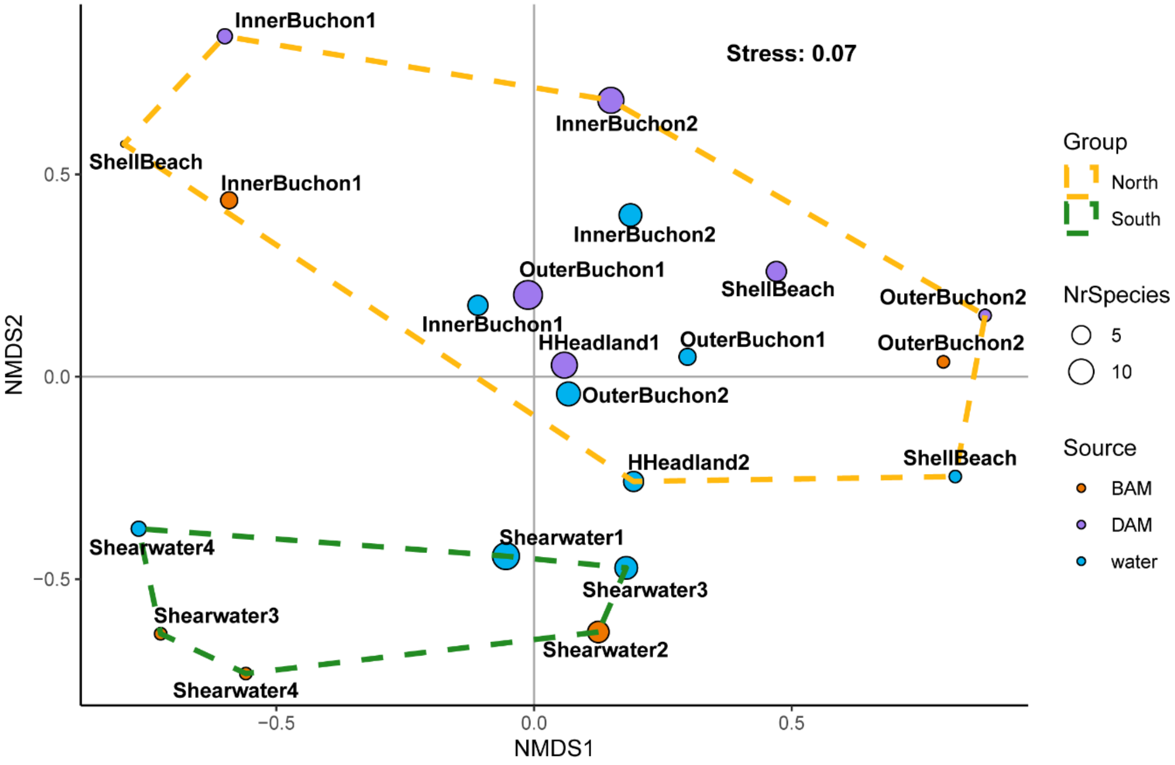

Figure S3 – Pattern of the species assemblages across sampling sites (considering only the offshore ones), as returned by the non-metric multidimensional scaling (nMDS) with Jaccard distance and based on presence/absence 12S eDNA data. Dots represent samples coloured according to the sampling methods. Polygons separate samples according to the biogeographic area, north and south *Humqaq*.

MOLECULAR ECOLOGY
RESOURCES

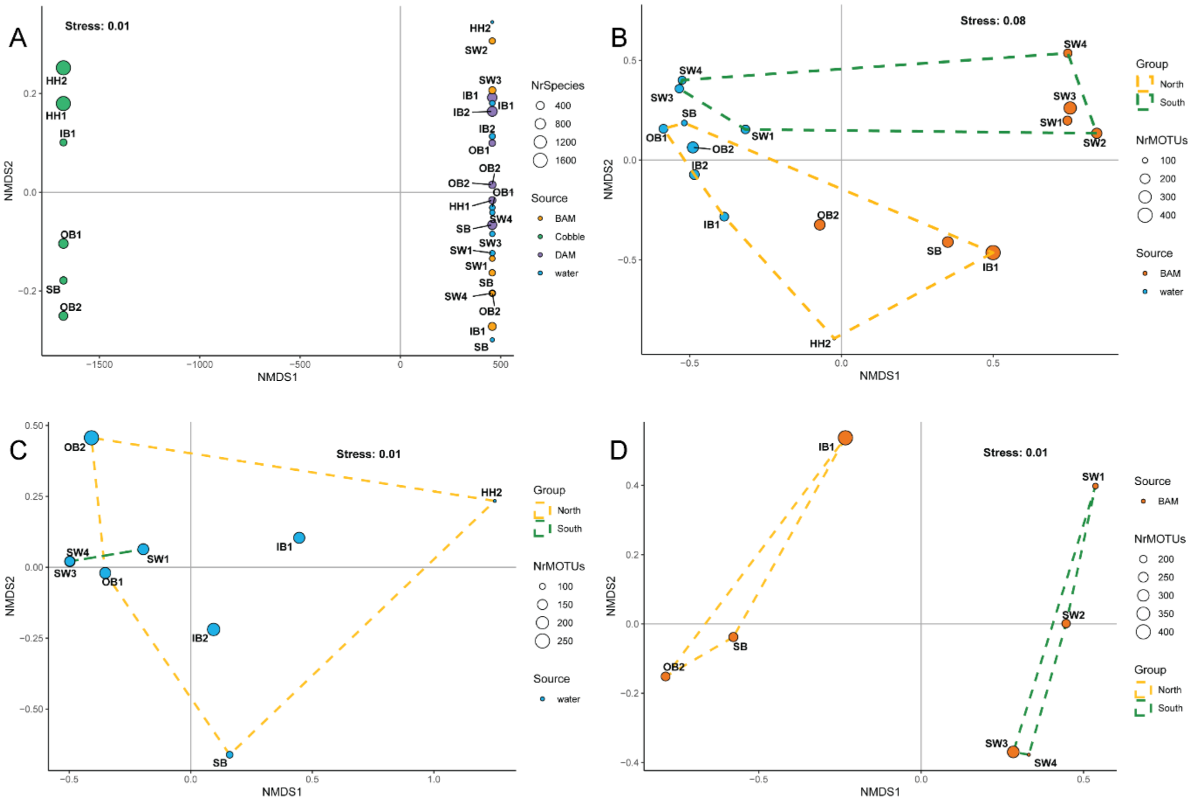

Figure S4 – Pattern of community composition across sampling sites as returned by the non-metric multidimensional scaling (nMDS) with Jaccard distance based on the presence/absence dataset including all MOTUs identified by COI. The ordination plots are based on (A) all COI data, (B) BAM and water, (C) only water and (C) only BAM samples. Dots represent samples and are coloured according to the sampling methods (i.e., BAM, cobbles, DAM and water). Polygons on graphs B, C and D separate samples according to the biogeographic area, north and south *Humqaq*.
